# Overview and evaluation of various frequentist test statistics using constrained statistical inference in the context of linear regression

**DOI:** 10.3389/fpsyg.2022.899165

**Published:** 2022-10-14

**Authors:** Caroline Keck, Axel Mayer, Yves Rosseel

**Affiliations:** ^1^Department of Data Analysis, Ghent University, Ghent, Belgium; ^2^Psychological Methods and Evaluation, Bielefeld University, Bielefeld, Germany

**Keywords:** informative hypothesis testing, constrained statistical inference, informative test statistics, type I error rates, naive mean squared error, corrected mean squared error, *F*^¯^-distribution, χ^¯2^-distribution

## Abstract

Within the framework of constrained statistical inference, we can test informative hypotheses, in which, for example, regression coefficients are constrained to have a certain direction or be in a specific order. A large amount of frequentist informative test statistics exist that each come with different versions, strengths and weaknesses. This paper gives an overview about these statistics, including the Wald, the LRT, the Score, the $\bar{F}$- and the *D*-statistic. Simulation studies are presented that clarify their performance in terms of type I and type II error rates under different conditions. Based on the results, it is recommended to use the Wald and $\bar{F}$-test rather than the LRT and Score test as the former need less computing time. Furthermore, it is favorable to use the degrees of freedom corrected rather than the naive mean squared error when calculating the test statistics as well as using the $\bar{F}$- rather than the ${\bar{\chi }}^{2}$-distribution when calculating the *p*-values.

## Introduction

Imagine a researcher wants to examine a novel psychotherapy program. A randomized experiment is set up with three treatment groups. One is a control group (*X* = 0), one participates in an established, standard psychotherapy program (*X* = 1) and one participates in the novel psychotherapy program (*X* = 2). No covariates are considered. The researcher is interested in the group means of the dependent variable *Y*, which denotes the score on a mental health questionnaire. Studies like this are usually conducted to show the superiority of the novel treatment over the standard treatment, as well as the superiority of the standard treatment over the control group. Thus, the researcher assumes that μ_2_ > μ_1_ > μ_0_. However, following classical null hypothesis testing procedures, we usually first test a hypothesis like *H*_0_ : μ_2_ = μ_1_ = μ_0_ against *H*_*a*_: not *H*_0_, that is, not all three means are equal. If we can reject *H*_0_ in favor of *H*_*a*_, a second step often follows, in which we execute pairwise comparisons to determine which means are equal and which means are not equal. This implies multiple testing, which brings along the risk of an inflated type I error rate. The framework of constrained statistical inference (Silvapulle and Sen, 2005; Hoijtink, 2012) allows us to test so-called informative hypotheses, meaning that we can test the null hypothesis *H*_0_ : μ_2_ = μ_1_ = μ_0_ against the ordered hypothesis *H*_*a*_ : μ_2_ > μ_1_ > μ_0_ in a single step. Thus, in contrast to classical null hypothesis testing, researchers have the advantages that they can formulate their hypotheses of interest directly, instead of making a detour via another hypothesis, while additionally avoiding to increase the risk for inflated type I error rates.

Informative hypothesis testing can be conducted by means of the Bayesian (see, e.g., Hoijtink et al., 2008; Hoijtink, 2012) as well as the frequentist (see, e.g., Barlow et al., 1972; Robertson et al., 1988; Silvapulle and Sen, 2005) approach, where the latter is the focus of this paper. The Bayesian approach is implemented in the R (R Core Team, 2020) package bain (Gu et al., 2020). The frequentist approach is implemented in SAS/STAT^®^ by means of the PLM procedure (for instructions, see Chapter 87 of SAS Institute Inc., 2015) as well as in several R packages including restriktor (Vanbrabant, 2020) and ic.infer (Grömping, 2010). Recent work of Keck et al. (2021) also demonstrated how to integrate informative hypothesis testing into the EffectLiteR (Mayer and Dietzfelbinger, 2019) package.

Restriktor and ic.infer use a broad range of test statistics, which are presented in Silvapulle and Sen (2005). However, research in the field of constrained statistical inference often uses the famous $\bar{F}$-statistic (see, e.g., Kuiper and Hoijtink, 2010; Vanbrabant et al., 2015) and neglects the distance statistic (*D*-statistic). Furthermore, each test statistic comes in various versions, for example depending on which estimate is used for the mean squared error or the variance-covariance matrix, and oftentimes, it is not obvious which software program uses which test statistic. There are also different options regarding the distributions that can be used to compute the *p*-values (${\bar{\chi }}^{2},\bar{F}$). At the same time, small sample properties of informative test statistics are mostly unknown. Finally, simulation studies that examine the performance of informative test statistics are lacking in the constrained statistical inference literature.

The aim of this paper is twofold. First, we want to give an overview of a broad range of different informative test statistics, including the Wald test, the likelihood-ratio test (LRT), the Score test, the $\bar{F}$- and the *D*-statistic as well as their different versions. Second, we want to clarify how those test statistics perform when sample and effect sizes, hypotheses and the distribution used for calculating the *p*-values vary. Note that we only consider the regression setting, where all variables are observed. The paper is structured as follows: We start by presenting the univariate linear regression model to explain all necessary terminology that is used in the following section, where we define the test statistics. These test statistics include “regular” as well as informative test statistics to illustrate the link between them. We also discuss different versions of these test statistics. Subsequently, we report about simulation studies that we conducted. We introduce the design of the studies, that included a broad range of sample sizes as well as effect sizes, and we outline type I and type II error rates. We conclude with a short discussion. Supplementary materials are provided and will be referenced throughout the paper.

## Univariate linear regression model

The univariate linear regression model for an observation *i* can be defined as:


(1) $$\begin{array}{l} y_{i}=\beta_{0}x_{i0}+\beta_{1}x_{i1}+\beta_{2}x_{i2}+...+\beta_{p}x_{ip}+\varepsilon_{i}=x_{i}^{\prime }\beta +\varepsilon_{i}, \end{array}$$

where *y*_*i*_ is the value of the response variable for observation *i* = 1, 2, …, *n*, *x*_*i*0_ is 1 and *x*_*i*1_, …, *x*_*ip*_ are the values of the *p* regressors for observation *i*, which are assumed to be fixed (in terms of repeated sampling). β_0_, …, β_*p*_ are the regression coefficients and ε_*i*_ is a residual for observation *i*. In matrix notation, the model can be written as ***y*** = ***X*****β** + ***ε***, where ***X*** is called the design matrix.

This regression model relies on several assumptions. First, we assume that the expected value of ε_*i*_ is zero. That is, *E*(ε_*i*_) = 0 for all *i*. In matrix notation, this is expressed as *E*(***ε***) = **0**, which implies that *E*(***y***) = ***X*****β**, meaning that there is a linear relationship between *E*(***y***) and the columns of ***X***. Second, we assume that *x*_*i*_ is non-stochastic and ***X*** is of full column rank. Third, we assume that the error term has a constant variance: $Var\left(\varepsilon_{i}\right)=\sigma_{\varepsilon }^{2}$ for all *i*. This implies that $Var\left(y_{i}\right)=\sigma_{\varepsilon }^{2}$ for all *i*. Fourth, we assume that the covariance of any two error terms is zero, that is *Cov*(ε_*i*_, ε_*j*_) = 0 for all (*i, j*), where *i* ≠ *j*.

The model can be estimated by means of different approaches such as ordinary least squares (OLS) or maximum likelihood (ML). It can be shown that under the presented assumptions, the OLS estimates of ***β*** are BLUE (best linear unbiased estimators, see, e.g., Seber and Lee, 2012). Using an example including four predictors, the following model is fitted:


(2) $$\begin{array}{l} y_{i}=\beta_{0}x_{i0}+\beta_{1}x_{i1}+\beta_{2}x_{i2}+\beta_{3}x_{i3}+\beta_{4}x_{i4}+\varepsilon_{i}, \end{array}$$

and ${\hat{\beta }}_{0},{\hat{\beta }}_{1},{\hat{\beta }}_{2},{\hat{\beta }}_{3}$, and ${\hat{\beta }}_{4}$ are obtained via OLS estimation. We may be interested in hypotheses concerning a single parameter like *H*_0_ : β_1_ = 0 vs. *H*_*a*_ : β_1_ ≠ 0 or we might be interested in hypotheses about nested model comparisons like *H*_0_ : β_1_ = 0 ∧ β_2_ = 0 vs. *H*_*a*_ : β_1_ ≠ 0 ∨ β_2_ ≠ 0 ∨ β_3_ ≠ 0 ∨ β_4_ ≠ 0. We can compute various important quantities that are used in hypothesis testing and that are characterized by a hat on top of it. Note that the hat indicates that estimation of the model parameters takes place in an unrestricted way, which will change once we test informative hypotheses. First, an unbiased estimator for the mean squared error is:


(3) $$\begin{array}{l} {\hat{\sigma }}_{\varepsilon }^{2}={Ŝ}_{corrected}^{2}=\frac{\hat{RSS}}{n-k}, \end{array}$$

where *k* is the column rank of ***X*** and $\hat{RSS}$ is the estimated residual sum of squares $\overset{n}{\underset{i=1}{\sum }}{ê}_{i}^{2}$, where ê_*i*_ = *y*_*i*_ − ŷ_*i*_ and ŷ_*i*_ are the model predicted values of the response variable. Note that by considering *k*, we yield a small-sample correction for the mean squared error, as opposed to simply using:


(4) $$\begin{array}{l} {Ŝ}_{naive}^{2}=\frac{\hat{RSS}}{n}, \end{array}$$

which corresponds to the maximum likelihood estimator of $\sigma_{\varepsilon }^{2}$.

The variance-covariance matrix of the estimated regression coefficients $\hat{\beta }$ is usually computed as:


(5) $$\begin{array}{l} VCOV\left(\hat{\beta }\right)=\frac{1}{n}{\hat{I}}_{1}^{-1}, \end{array}$$

where $\hat{I}$_1_ is the unit information matrix:


(6) $$\begin{array}{l} {\hat{I}}_{1}=\frac{1}{n\;{Ŝ}_{corrected}^{2}}X^{\prime }X. \end{array}$$

Note that if certain model assumptions are violated, for example if the error term does not have a constant variance, robust versions of the standard errors (Huber, 1967; White, 1980) and the variance-covariance matrix (Zeileis, 2006) can be used.

We can also test hypotheses about linear or non-linear combinations of regression parameters, like *H*_0_ : β_1_ + β_2_ = 0 ∧ β_3_ + β_4_ = 0 vs. *H*_*a*_ : β_1_ + β_2_ ≠ 0 ∨ β_3_ + β_4_ ≠ 0. Note that in this paper, we will focus only on hypotheses containing linear combinations of regression coefficients. These combinations are specified by means of the ***R***-matrix and each part of the hypothesis can be expressed as a row in ***R***:


(7) $$\begin{array}{l} r_{1}^{\prime }=\left(\begin{array}{ccccc} 0 & 1 & 1 & 0 & 0 \end{array}\right), \end{array}$$

(8) $$\begin{array}{l} r_{2}^{\prime }=\left(\begin{array}{ccccc} 0 & 0 & 0 & 1 & 1 \end{array}\right), \end{array}$$

leading to the full constraint matrix:


(9) $$\begin{array}{l} R=\left(\begin{array}{ccccc} 0 & 1 & 1 & 0 & 0\\ 0 & 0 & 0 & 1 & 1 \end{array}\right). \end{array}$$

Then the hypothesis of interest can be expressed as *H*_0_ : ***R*****β** = **0** vs. *H*_*a*_ : ***R*****β** ≠ **0**. Note that all kinds of hypotheses, including the single parameter case and comparisons of nested models, as discussed before, can be expressed by means of the ***R***-matrix.

In case our hypothesis of interest contains inequality constraints, like *H*_*a*_ : β_1_ + β_2_ > 0∨ β_3_ + β_4_ > 0, ***R*** still looks the same, but we need to fit a model where we enforce the inequality constraints on the regression coefficients. This can be done by means of quadratic programming, for example using the subroutine solve.QP() of the R package quadprog (Turlach and Weingessel, 2019). It implements the dual method of Goldfarb and Idnani (1982, 1983). If we apply this method in the linear regression context, it has the following form (see “Data Sheet 1” in the Supplementary materials for further explanations):


(10) $$\begin{array}{l} min\left(-y^{\prime }X\beta +\frac{1}{2}\beta^{\prime }X^{\prime }X\beta \right)\text{ with the constraints }R\beta \geq \beta_{0}. \end{array}$$

Note that all quantities based on an inequality constrained model are denoted by a tilde on top of them. Assume that the unconstrained estimates ${\hat{\beta }}^{\prime }$ are (0.100 −*0.130*0.100 −*0.240*0.250), but the inequality constrained estimates ${\tilde{\beta }}^{\prime }$ may be (0.110 −*0.110*0.120 −*0.230*0.240), where the estimates of β_0_, β_3_ and β_4_ may also change slightly, even though they already satisfied the constraints in the unrestricted estimation. The restricted estimation will also lead to different residuals than the unrestricted estimation.

If our hypothesis of interest contains equality constraints, for example *H*_*a*_ : β_1_ + β_2_ = 0 ∨ β_3_ + β_4_ = 0, the equality constrained estimates $\bar{\beta }$ can also be found via quadratic programming. Note that here, *H*_*a*_ from informative hypothesis testing equals *H*_0_ from classical null hypothesis testing. Similarly, all estimated quantities with a bar on top are both the quantities from the equality constrained fit in informative hypothesis testing and the quantities obtained based on *H*_0_ in classical null hypothesis testing, which are in fact equality constrained estimates as well. The corresponding mean squared error terms for the inequality and equality constrained case are defined as follows:


(11) $$\begin{array}{l} {\tilde{S}}_{corrected}^{2}=\frac{\tilde{RSS}}{n-k}\;, \end{array}$$

(12) $$\begin{array}{l} {\tilde{S}}_{naive}^{2}=\frac{\tilde{RSS}}{n}\;, \end{array}$$

(13) $$\begin{array}{l} {\bar{S}}_{corrected}^{2}=\frac{\bar{RSS}}{n-\left(k-h\right)}\;, \end{array}$$

(14) $$\begin{array}{l} {\bar{S}}_{naive}^{2}=\frac{\bar{RSS}}{n}\;, \end{array}$$

where $\tilde{RSS}$ is the residual sum of squares of the inequality constrained fit $\overset{n}{\underset{i=1}{\sum }}{ẽ}_{i}^{2}$, where ẽ_*i*_ = *y*_*i*_ − ỹ_*i*_ and ỹ_*i*_ are the model predicted values of the response variable. Furthermore, $\bar{RSS}$ is the residual sum of squares under the equality constrained fit $\overset{n}{\underset{i=1}{\sum }}{ē}_{i}^{2}$, where ē_*i*_ = *y*_*i*_ − ȳ_*i*_ and ȳ_*i*_ are the model predicted values of the response variable. Finally, *h* is the row rank of ***R***.

Similarly, we can define the unit information matrices of the inequality and equality constrained fits:


(15) $$\begin{array}{l} {\tilde{I}}_{1}=\frac{1}{n\;{\tilde{S}}_{corrected}^{2}}X^{\prime }X, \end{array}$$

(16) $$\begin{array}{l} {\bar{I}}_{1}=\frac{1}{n\;{\bar{S}}_{corrected}^{2}}X^{\prime }X. \end{array}$$

Note that ***X*** from the inequality constrained fit equals ***X*** from the unconstrained fit. The estimates $\hat{\beta },\tilde{\beta }$ and $\bar{\beta }$ as well as the corresponding mean squared error terms and unit information matrices are used in the test statistics that are presented in the subsequent section.

## Hypothesis testing

In order to give a broad overview about different test statistics, we present regular test statistics used in classical null hypothesis testing, as well as informative test statistics used in informative hypothesis testing. Note that an overview table containing all test statistics is provided at the end of each section. All test statistics can be applied in the setting of linear regression. “Data Sheet 2” in the Supplementary materials shows how these test statistics are implemented in R code.

### Classical null hypothesis testing

The test statistics from classical null hypothesis testing that we will explain include the Wald test, the LRT, the Score test, the *F*-test as well as the *t*-test. The large sample test statistics, that is the Wald test, the LRT and the Score test, can be defined as follows Buse (1982):


(17) $$\begin{array}{l} Wald=n\left(R\hat{\beta }\right)\prime (R{\hat{I}}_{1}^{-1}R^{\prime }{)}^{-1}\left(R\hat{\beta }\right), \end{array}$$

(18) $$\begin{array}{l} LRT=-2\cdot \left[\ell \left(\bar{\beta }\right)-\ell \left(\hat{\beta }\right)\right], \end{array}$$

(19) $$\begin{array}{l} Score=\frac{1}{n}S{\left(\bar{\beta }\right)}^{\prime }{\bar{I}}_{1}^{-1}S\left(\bar{\beta }\right), \end{array}$$

where $\ell \left(\bar{\beta }\right)$ is the log-likelihood evaluated at $\bar{\beta }$, $\ell \left(\hat{\beta }\right)$ is the log-likelihood evaluated at $\hat{\beta }$ and $S\left(\bar{\beta }\right)=\frac{\partial }{\partial \bar{\beta }}\ell \left(\bar{\beta }\right)$ is the score function evaluated at $\bar{\beta }$. All three test statistics follow asymptotically a χ^2^-distribution under the null hypothesis with *df* = *h*, if the model is correct.

Note that all three test statistics implicitly depend on *S*^2^ in the information matrices (see Equation 6) and in the log-likelihoods. In the regression setting, since we always know what the residual degrees of freedom are, we can use ${Ŝ}_{corrected}^{2}$ instead of ${Ŝ}_{naive}^{2}$ to obtain the corrected instead of naive test statistic versions. That way, we can use the *F*-distribution with *df*_1_ = *h, df*_2_ = *n* − *p* to obtain the *p*-values, which is more precise in small samples compared to the χ^2^-distribution.

Note that the LRT, the Wald and the Score test are asymptotically equivalent. However, it has been shown that the values of the Wald test are always slightly larger than the values of the LRT, which in turn are always slightly larger than the values of the Score test (Buse, 1982, p. 157). Thus, using the same critical χ^2^ value, the tests may have different power properties, which can be one aspect guiding the choice between them. Another aspect may be the time it takes to compute the three tests. For the Wald test, we need to fit the unconstrained model, whereas for the Score test, we need to fit the equality constrained model and for the LRT, we need to fit both the unconstrained and equality constrained model. In many cases, fitting the unconstrained model takes the least amount of time, which is why the Wald test is chosen often. However, in some cases, for example if the equality constrained model has a lot less parameters than the unconstrained model, it may be faster to fit the equality constrained model compared to the unconstrained model.

The *F*-test can be calculated as Seber and Lee (2012, p. 100):


(20) $$\begin{array}{l} F_{corrected}=\frac{\frac{1}{h}\left[\bar{RSS}-\hat{RSS}\right]}{{Ŝ}_{corrected}^{2}}. \end{array}$$

Another test statistic version results from using ${Ŝ}_{naive}^{2}$ instead of ${Ŝ}_{corrected}^{2}$, which we denote as *F*_*naive*_. Seber and Lee (2012, p. 100) show that *F*_*corrected*_ can be re-written to contain the unit information matrix:


(21) $$\begin{array}{l} F_{corrected}^{info}=\frac{n}{h}\left(R\hat{\beta }\right)\prime (R{\hat{I}}_{1}^{-1}R^{\prime }{)}^{-1}\left(R\hat{\beta }\right), \end{array}$$

where the superscript “info” refers to the information matrix. When ${Ŝ}_{naive}^{2}$ instead of ${Ŝ}_{corrected}^{2}$ is used in constructing the unit information matrix, we call this test statistic $F_{naive}^{info}$. If the model is specified correctly, *F*_*corrected*_ follows an *F*-distribution with *df*_1_ = *h, df*_2_ = *n* − *k* under the null hypothesis.

The one-sample *t*-test is defined as Allen(1997, p. 67):


(22) $$\begin{array}{l} t=\frac{\hat{\beta }-\bar{\beta }}{SE_{\hat{\beta }}}, \end{array}$$

where $\bar{\beta }$ is the value of β under the null hypothesis and $SE_{\hat{\beta }}$ is the standard error of $\hat{\beta }$. Under the null hypothesis, *t* is *t*-distributed with *df* = *n* − *k*, if the model is correct. Note that if *h* = 1 the *t*- and *F*-statistic are related in a certain way, which is *t*^2^ = *F*.

It is widely known that the one-sample *t*-test can be used for testing both two-sided hypotheses like *H*_0_ : β = 0 against *H*_*a*_ : β ≠ 0 as well as one-sided hypotheses like *H*_0_ : β = 0 against *H*_*a*_ : β > 0 or *H*_*a*_ : β < 0. The test statistic stays the same in both cases, but the *p*-value is computed differently. That is, when testing a two-sided hypothesis, half of the significance level is allocated to each side of the *t*-distribution, whereas when testing a one-sided hypothesis, all of it is allocated to one side of the *t*-distribution. That means that the cut-off levels, denoting from which point on the *t*-statistic can be considered to be significant, change. The two-sided *p*-value, which is the default output of most statistical software, simply adds up the probabilities of the negative and positive version of the observed *t*-value (*t*_*obs*_), independently of whether it was in fact positive or negative:


(23) $$\begin{array}{l} p_{two-sided}=2\cdot P(t>|t_{obs}|)\\ \;=P(t>t_{obs})+P(t<-t_{obs}). \end{array}$$

Since the *t*-distribution is symmetric, *P*(*t* > *t*_*obs*_) is the same as *P*(*t* < −*t*_*obs*_). When we are interested in the one-sided *p*-value and *H*_*a*_ : β > 0, the *p*-value is obtained as:


(24) $$\begin{array}{l} p_{one-sided}=P\left(t>t_{obs}\right), \end{array}$$

whereas if *H*_*a*_ : β < 0, the *p*-value is obtained as:


(25) $$\begin{array}{l} p_{one-sided}=P\left(t<t_{obs}\right). \end{array}$$

Note that in case the obtained *t*-value is a positive number and we are interested in *H*_*a*_ : β > 0 or in case *t* is a negative number and we are interested in *H*_*a*_ : β < 0, the one-sided *p*-value can be obtained by dividing the two-sided *p*-value by 2.

In summary, the *t*-statistic is a special case, since this statistic from the classical null hypothesis testing framework can be used for testing an informative hypothesis, as long as the hypothesis only contains one parameter. If we are interested in more than one parameter, we can no longer use the *t*-statistic, but have to use an informative test statistic. Table 1 shows an overview about all presented regular test statistics.

**Table 1:** Overview of all presented regular test statistics.

**Regular test statistics**	**Formula**
*LRT*_*naive*/*corrected*_	$-2\cdot \left[\ell \left(\bar{\beta }\right)-\ell \left(\hat{\beta }\right)\right]$
*Wald*_*naive*/*corrected*_	$n{\left(R\hat{\beta }\right)}^{\prime }{\left(R{\hat{I}}_{1}^{-1}R^{\prime }\right)}^{-1}\left(R\hat{\beta }\right)$
*Score*_*naive*/*corrected*_	$\frac{1}{n}S{\left(\bar{\beta }\right)}^{\prime }{\bar{I}}_{1}^{-1}S\left(\bar{\beta }\right)$
*F*_*naive*/*corrected*_	$\frac{\frac{1}{h}\left[\bar{RSS}-\hat{RSS}\right]}{{Ŝ}_{naive/corrected}^{2}}$
*t*	$\frac{\hat{\beta }-\bar{\beta }}{SE_{\hat{\beta }}}$

### Informative hypothesis testing

Informative test statistics are often a modified version of the regular test statistics. In case the model is correct, the large sample informative test statistics, including the LRT, the Wald test, the Score test and the *D*-statistic, asymptotically follow a ${\bar{\chi }}^{2}$-distribution under the null hypothesis, which is a mixture of χ^2^-distributions. The small sample informative test statistic, that is the $\bar{F}$-statistic, follows an $\bar{F}$-distribution under the null hypothesis, if the model is correctly specified. The $\bar{F}$-distribution is a mixture of *F*-distributions. Note that similar to classical null hypothesis testing, we can use the corrected instead of naive mean squared error to obtain the large sample test statistics. In that way, we can calculate the *p*-values by means of the $\bar{F}$-distribution instead of the ${\bar{\chi }}^{2}$-distribution to obtain more precise results in small sample sizes.

The *LRT*_*corrected*_ test statistic can be calculated as follows Silvapulle and Sen (2005, p. 157):


(26) $$\begin{array}{l} LRT_{corrected}=-2\cdot \left[\ell \left(\bar{\beta }\right)-\ell \left(\tilde{\beta }\right)\right], \end{array}$$

where $\ell \left(\bar{\beta }\right)$ is the log-likelihood evaluated at $\bar{\beta }$ and $\ell \left(\tilde{\beta }\right)$ is the log-likelihood evaluated at $\tilde{\beta }$. $\ell \left(\bar{\beta }\right)$ has been calculated using ${\bar{S}}_{corrected}^{2}$ and $\ell \left(\tilde{\beta }\right)$ has been calculated using ${\tilde{S}}_{corrected}^{2}$. If ${\bar{S}}_{naive}^{2}$ and ${\tilde{S}}_{naive}^{2}$ were used instead, we would obtain *LRT*_*naive*_.

The Wald statistic can be found in Silvapulle and Sen (2005, p. 154):


(27) $$\begin{array}{l} Wald_{corrected}^{info}=\frac{n}{{Ŝ}_{corrected}^{2}}{\left(R\tilde{\beta }\right)}^{\prime }{\left(RW^{-1}R^{\prime }\right)}^{-1}\left(R\tilde{\beta }\right), \end{array}$$

where $W=\frac{1}{n}X^{\prime }X$. The Wald version where we use ${Ŝ}_{naive}^{2}$ instead of ${Ŝ}_{corrected}^{2}$ is called $Wald_{naive}^{info}$. Both versions implicitly contain $\hat{I}$_1_ (see Equation 6), which can also be replaced by $\tilde{I}$_1_. Note that $Wald_{naive}^{info}$ will give different results, especially in small sample sizes, due to the missing correction. Assuming $VCOV\left(\hat{\beta }\right)$ is defined as in Equation 5, we can re-write the Wald statistic as:


(28) $$\begin{array}{ll} Wald^{VCOV} & ={\left[R\tilde{\beta }\right]}^{\prime }{\left[RVCOV\left(\hat{\beta }\right)R^{\prime }\right]}^{-1}\left[R\tilde{\beta }\right], \end{array}$$

which is identical to $Wald_{corrected}^{info}$. Note that we can also replace $VCOV\left(\hat{\beta }\right)$ by a more robust sandwich-estimator, which is not commonly done in the applied literature.

The *D*-statistic is calculated as follows (Silvapulle and Sen, 2005, p. 159):


(29) $$\begin{array}{l} D_{corrected}=\frac{2\cdot n}{{Ŝ}_{corrected}^{2}}\left[d\left(\bar{\beta }\right)-d\left(\tilde{\beta }\right)\right], \end{array}$$

where $d\left(\bar{\beta }\right)$ and $d\left(\tilde{\beta }\right)$ are the values of the following two functions at their solutions (see “Data Sheet 3” in the Supplementary materials for further information):


(30) $$\begin{array}{l} f\left(\beta \right)={\left(\hat{\beta }-\beta \right)}^{\prime }W\left(\hat{\beta }-\beta \right)\text{ under the constraint }R\beta =0, \end{array}$$

(31) $$\begin{array}{l} f\left(\beta \right)={\left(\hat{\beta }-\beta \right)}^{\prime }W\left(\hat{\beta }-\beta \right)\text{ under the constraint }R\beta \geq 0. \end{array}$$

When minimizing these functions, we treat $\hat{\beta }$ and ***W*** as known constants. Note that in the regression case, *D*_*corrected*_ is identical to $Wald_{corrected}^{info}$ and *Wald*^*VCOV*^, as long as ${Ŝ}_{corrected}^{2}$ is used. In contrast, if we switch to using ${Ŝ}_{naive}^{2}$, we obtain *D*_*naive*_, in which case $D_{naive}=Wald_{naive}^{info}$.

The $\bar{F}$-statistic can be found in (Silvapulle and Sen, 2005, p. 29):


(32) $$\begin{array}{l} {\bar{F}}_{corrected}=\frac{\bar{RSS}-\tilde{RSS}}{{Ŝ}_{corrected}^{2}}. \end{array}$$

According to Silvapulle and Sen (2005, p. 29), including the constant $\frac{1}{h}$ from the regular *F*-statistic in the $\bar{F}$-statistic is not necessary, as it does not affect the results. Again, when using ${Ŝ}_{naive}^{2}$ instead of ${Ŝ}_{corrected}^{2}$, we obtain ${\bar{F}}_{naive}$. We can re-write the $\bar{F}$-statistic similarly to how we re-wrote the *F*-statistic. Assuming that we use ${Ŝ}_{corrected}^{2}$ to compute the unit information matrix, we obtain:


(33) $$\begin{array}{l} {\bar{F}}_{corrected}^{info}=n{\left(R\tilde{\beta }\right)}^{\prime }(R{\hat{I}}_{1}^{-1}R^{\prime }{)}^{-1}\left(R\tilde{\beta }\right). \end{array}$$

Again, $\hat{I}$_1_ can be replaced by $\tilde{I}$_1_.

There are various versions of the Score statistic. $Score_{corrected}^{U}$ can be found in Silvapulle and Sen (2005, p. 159):


(34) $$\begin{array}{l} Score_{corrected}^{U}=\frac{1}{n\cdot {Ŝ}_{corrected}^{2}}U^{\prime }{\left(RW^{-1}R^{\prime }\right)}^{-1}U, \end{array}$$

where $U=RW^{-1}\left[S\left(\tilde{\beta }\right)-S\left(\bar{\beta }\right)\right]$. When using ${Ŝ}_{naive}^{2}$ as compared to ${Ŝ}_{corrected}^{2}$, we obtain $Score_{naive}^{U}$. Another version of the Score statistic, $Score_{corrected}^{null-info}$, is defined as follows Silvapulle and Silvapulle (1995, p. 342):


(35) $$\begin{array}{l} Score_{corrected}^{null-info}=\frac{1}{n}{\left[S\left(\bar{\beta }\right)-S\left(\tilde{\beta }\right)\right]}^{\prime }{\bar{I}}_{1}^{-1}\left[S\left(\bar{\beta }\right)-S\left(\tilde{\beta }\right)\right], \end{array}$$

where $\bar{I}$_1_ has been calculated by means of ${\bar{S}}_{corrected}^{2}$ (see Equation 13). In contrast, if we use ${\bar{S}}_{naive}^{2}$, we obtain $Score_{naive}^{null-info}$.

Furthermore, $Score_{corrected}^{info}$ can be calculated as Silvapulle and Sen (2005, p. 166):


(36) $$\begin{array}{l} Score_{corrected}^{info}=\frac{1}{n}P^{\prime }(R{\hat{I}}_{1}^{-1}R^{\prime }{)}^{-1}P, \end{array}$$

where $P=R{\hat{I}}_{1}^{-1}\left[S\left(\tilde{\beta }\right)-S\left(\bar{\beta }\right)\right]$ and $\hat{I}$_1_ is calculated using ${Ŝ}_{corrected}^{2}$ and can be replaced by either $\tilde{I}$_1_ or $\bar{I}$_1_. If we use ${Ŝ}_{naive}^{2}$ to calculate $\hat{I}$_1_, we obtain $Score_{naive}^{info}$. Silvapulle and Sen (2005, p. 166) mention another way to express $Score_{corrected}^{info}$:


(37) $$\begin{array}{l} Score_{corrected}^{info,Robertson}=\frac{1}{n}{\left[S\left(\tilde{\beta }\right)-S\left(\bar{\beta }\right)\right]}^{\prime }{\hat{I}}_{1}^{-1}\left[S\left(\tilde{\beta }\right)-S\left(\bar{\beta }\right)\right], \end{array}$$

where the superscript “Robertson” indicates that this is the version defined by Robertson et al. (1988), $\hat{I}$_1_ is calculated using ${Ŝ}_{corrected}^{2}$ and can be replaced by either $\tilde{I}$_1_ or $\bar{I}$_1_. Assuming that $VCOV\left(\hat{\beta }\right)$ is defined as in Equation 5, $Score_{corrected}^{info}$ can be re-written as:


(38) $$\begin{array}{l} Score^{VCOV}=V^{\prime }{\left[RVCOV\left(\hat{\beta }\right)R^{\prime }\right]}^{-1}V, \end{array}$$

where $V=RVCOV\left(\hat{\beta }\right)\left[S\left(\tilde{\beta }\right)-S\left(\bar{\beta }\right)\right]$, again allowing for a more robust sandwich-estimator of $VCOV\left(\hat{\beta }\right)$ to be inserted. Table 2 gives an overview about all the informative test statistics that were presented.

**Table 2:** Overview of all presented informative test statistics.

**Informative test statistics**	**Formulas**
*LRT*_*naive*/*corrected*_	$-2\cdot \left[\ell \left(\bar{\beta }\right)-\ell \left(\tilde{\beta }\right)\right]$
$Wald_{naive}^{info}$	$\frac{n}{{Ŝ}_{naive}^{2}}{\left(R\tilde{\beta }\right)}^{\prime }{\left(RW^{-1}R^{\prime }\right)}^{-1}\left(R\tilde{\beta }\right)$
$Wald_{corrected}^{info}=Wald^{VCOV}$	$\frac{n}{{Ŝ}_{corrected}^{2}}{\left(R\tilde{\beta }\right)}^{\prime }{\left(RW^{-1}R^{\prime }\right)}^{-1}\left(R\tilde{\beta }\right)$
	$={\left[R\tilde{\beta }\right]}^{\prime }{\left[RVCOV\left(\hat{\beta }\right)R^{\prime }\right]}^{-1}\left[R\tilde{\beta }\right]$
*D*_*naive*/*corrected*_	$\frac{2\cdot n}{{Ŝ}_{naive/corrected}^{2}}\left[d\left(\bar{\beta }\right)-d\left(\tilde{\beta }\right)\right]$
${\bar{F}}_{naive}$	$\frac{\bar{RSS}-\tilde{RSS}}{{Ŝ}_{naive}^{2}}$
${\bar{F}}_{corrected}={\bar{F}}_{corrected}^{info}$	$\frac{\bar{RSS}-\tilde{RSS}}{{Ŝ}_{corrected}^{2}}$
	$=n{\left(R\tilde{\beta }\right)}^{\prime }{\left(R{\hat{I}}_{1}^{-1}R^{\prime }\right)}^{-1}\left(R\tilde{\beta }\right)$
$Score_{naive/corrected}^{U}$	$\frac{1}{n\cdot {Ŝ}_{naive/corrected}^{2}}U^{\prime }{\left(RW^{-1}R^{\prime }\right)}^{-1}U$
$Score_{naive/corrected}^{null-info}$	$\frac{1}{n}{\left[S\left(\bar{\beta }\right)-S\left(\tilde{\beta }\right)\right]}^{\prime }{\bar{I}}_{1}^{-1}\left[S\left(\bar{\beta }\right)-S\left(\tilde{\beta }\right)\right]$
$Score_{naive}^{info}=Score_{naive}^{info,Robertson}$	$\frac{1}{n}P^{\prime }{\left(R{\hat{I}}_{1}^{-1}R^{\prime }\right)}^{-1}P$
	$=\frac{1}{n}{\left[S\left(\tilde{\beta }\right)-S\left(\bar{\beta }\right)\right]}^{\prime }{\hat{I}}_{1}^{-1}\left[S\left(\tilde{\beta }\right)-S\left(\bar{\beta }\right)\right]$
$Score_{corrected}^{info}=Score_{corrected}^{info,Robertson}$ = *Score*^*VCOV*^	$\frac{1}{n}P^{\prime }{\left(R{\hat{I}}_{1}^{-1}R^{\prime }\right)}^{-1}P$
	$=\frac{1}{n}{\left[S\left(\tilde{\beta }\right)-S\left(\bar{\beta }\right)\right]}^{\prime }{\hat{I}}_{1}^{-1}\left[S\left(\tilde{\beta }\right)-S\left(\bar{\beta }\right)\right]$
	$=V^{\prime }{\left[RVCOV\left(\hat{\beta }\right)R^{\prime }\right]}^{-1}V$

#### *P*-values

There are two approaches for calculating the *p*-value of informative test statistics (Silvapulle and Sen, 2005). In this paper, we use the approach where we first calculate the weights of the respective mixture distribution (${\bar{\chi }}^{2},\bar{F}$). Note that the sum of the weights from 0 to *q* is one, where *q* is the rank of ***X*** under the null hypothesis.

If the residuals of our data are normally distributed, we can use the multivariate normal probability function as well as the ic.weight() function of the R package ic.infer (Grömping, 2010) to compute the weights. These calculations are also implemented in the R package restriktor (Vanbrabant, 2020). Once we have computed the weights, the *p*-values of the observed ${\bar{\chi }}^{2}$-value (${\bar{\chi }}_{obs}^{2}$) and of the observed $\bar{F}$-value (${\bar{F}}_{obs}$) are obtained as follows Silvapulle and Sen (2005, pp. 86 and 99):


(39) $$\begin{array}{l} Pr\left({\bar{\chi }}^{2}\geq {\bar{\chi }}_{obs}^{2}\right)=\overset{q}{\underset{i=0}{\sum }}w_{i}\left(H_{0},H_{a}\right)\text{Pr}\left[\left(h-q+i\right)\chi_{h-q+i}^{2}\geq {\bar{\chi }}_{obs}^{2}\right], \end{array}$$

(40) $$\begin{array}{l} Pr\left(\bar{F}\geq {\bar{F}}_{obs}\right)=\overset{q}{\underset{i=0}{\sum }}w_{i}\left(H_{0},H_{a}\right)\text{Pr}\left[\left(h-q+i\right)F_{h-q+i,n-p}\geq {\bar{F}}_{obs}\right]. \end{array}$$

It can be expected that the *p*-values are very similar, irrespective of whether they are calculated based on the ${\bar{\chi }}^{2}$- or $\bar{F}$-distribution, as long as sample sizes are large. However, for small sample sizes, the $\bar{F}$-distribution should yield more accurate results.

## Simulation studies

We conducted several simulation studies to examine the impact of different conditions on the performance of the presented test statistics in terms of type I and type II error rates. We were interested in the effects of sample and effect sizes, the number of regression parameters considered in *H*_*a*_ as well as the distribution used for calculating the *p*-values. Our main motivation was to provide a reference framework for applied researchers who wish to test informative hypotheses, helping them to chose the optimal test statistic(s) in the present situation.

### Design

We generated a design matrix ***X***, including data for five regression coefficients $\beta^{\prime }=\left(\beta_{1}\;\beta_{2}\;\beta_{3}\;\beta_{4}\;\beta_{5}\right)$ and considered effect sizes of *f*^2^ = 0.02 (small), *f*^2^ = 0.10 (medium) and *f*^2^ = 0.35 (large) and sample sizes of 10, 25, 50, 100, 500, 1000, 2000, and 10000. For examining the type I error rate, we generated a random outcome *Y*, whereas for examining the type II error rate, we fixed all βs to 0.1 and generated *y* with a random error term that was specific for the effect size used. Since $f^{2}=\frac{R^{2}}{1-R^{2}}$, where *R*^2^ is the determination coefficient, we can calculate the error terms of *y* by plugging in the *f*^2^-specific value of *R*^2^ in


(41) $$\begin{array}{l} S_{y}^{2}=\left[\beta Cor\left(X\right)\beta \right]\times \frac{1-R^{2}}{R^{2}}, \end{array}$$

where *Cor*(***X***) is the correlation matrix of the design matrix ***X***. The number of replications was 1000.

We considered two different kinds of ***R*** matrices, where the first one was defined as follows:


(42) $$\begin{array}{l} R_{1}=\left(\begin{array}{cccccc} 0 & 1 & 0 & 0 & 0 & 0 \end{array}\right). \end{array}$$

This represents the hypothesis that only β_1_ is greater than zero: *H*_*a*_ : β_1_ > 0. The second ***R*** matrix was defined as:


(43) $$\begin{array}{l} R_{2}=\left(\begin{array}{cccccc} 0 & 1 & 0 & 0 & 0 & 0\\ 0 & 0 & 1 & 0 & 0 & 0\\ 0 & 0 & 0 & 1 & 0 & 0\\ 0 & 0 & 0 & 0 & 1 & 0\\ 0 & 0 & 0 & 0 & 0 & 1 \end{array}\right), \end{array}$$

stating that at least one of the regression coefficients, except the intercept, are greater than zero: *H*_*a*_ : β_1_ > 0 ∨ β_2_ > 0 ∨ β_3_ > 0 ∨ β_4_ > 0 ∨ β_5_ > 0.

To compute the test statistics, we used ${Ŝ}_{naive}^{2}$ and ${Ŝ}_{corrected}^{2}$ as well as ${\tilde{S}}_{naive}^{2},{\tilde{S}}_{corrected}^{2},{\bar{S}}_{naive}^{2}$ and ${\bar{S}}_{corrected}^{2}$ and to compute the *p*-values, we used the ${\bar{\chi }}^{2}$- as well as the $\bar{F}$-distribution. In addition to the manual calculation of the test statistics, we also included the test statistics as reported by the R package restriktor.

### Type I results

Test statistics were first applied the way they are presented in the referenced literature. That is, $Wald_{naive}^{info}$ makes use of ${Ŝ}_{naive}^{2}$, whereas all other test statistics make use of ${Ŝ}_{corrected}^{2}$ (or ${\tilde{S}}_{corrected}^{2},{\bar{S}}_{corrected}^{2}$). For calculating the *p*-values, the ${\bar{\chi }}^{2}$-distribution is used for *LRT*_*corrected*_, $Wald_{naive}^{info}$, $Wald_{corrected}^{info}$, *Wald*^*VCOV*^, *D*_*corrected*_, $Score_{corrected}^{U}$, $Score_{corrected}^{null-info}$, $Score_{corrected}^{info}$ and *Score*^*VCOV*^. The $\bar{F}$-distribution is used for calculating the *p*-values for the $\bar{F}$-statistic, the *F*-distribution is used for calculating the *p*-values for the *F*-statistic and the *t*-distribution is used for calculating the *p*-values for the *t*-statistic. Note that restriktor always uses ${Ŝ}_{corrected}^{2}$ (or ${\tilde{S}}_{corrected}^{2},{\bar{S}}_{corrected}^{2}$) for all available test statistics and always calculates the *p*-value based on the $\bar{F}$-distribution. Tables 3, 4 show the results.

**Table 3:** Type I error rates when using ***R***_1_ and applying the test statistics as outlined in the referenced books.

				$Wald_{corr.}^{info}$								
***n***	***LRT*_*corr*._**	***LRT*_*restr*._**	$Wald_{naive}^{info}$	***Wald* ^*VCOV*^**	$Score_{corr.}^{U}$	$Score_{corr.}^{null-info}$	$Score_{restr.}^{null-info}$	$Score_{corr.}^{info}$	${\bar{F}}_{corr.}$	***F*_*corr*._**	***t*_*one*−*s*._**	***t*_*two*−*s*._**
				***D*_*corr*._**				***Score* ^*VCOV*^**	${\bar{F}}_{restr.}$			
10000	0.047	0.047	0.047	0.047	0.047	0.047	0.047	0.047	0.047	0.050	0.047	0.050
2000	0.054	0.054	0.054	0.054	0.057	0.054	0.054	0.054	0.054	0.060	0.054	0.060
1000	0.057	0.057	0.058	0.057	0.058	0.057	0.057	0.057	0.057	0.067	0.057	0.067
500	0.058	0.058	0.058	0.055	0.053	0.055	0.055	0.055	0.055	0.049	0.055	0.049
100	0.054	0.054	0.056	0.051	0.054	0.050	0.048	0.048	0.049	0.043	0.049	0.043
50	0.057	0.058	0.060	0.054	**0.066**	0.051	0.044	0.044	0.049	0.044	0.049	0.044
25	**0.074**	**0.074**	**0.092**	**0.066**	**0.089**	0.057	0.047	0.045	0.057	0.057	0.057	0.057
10	**0.125**	**0.112**	**0.186**	**0.098**	**0.169**	0.054	0.002	0.000	0.054	**0.061**	0.054	**0.061**

The test statistics are abbreviated as follows: *LRT*_*corrected*_ as *LRT*_*corr*._, *LRT*_*restriktor*_ as *LRT*_*restr*._, $Wald_{corrected}^{info}$ as $Wald_{corr.}^{info}$, *D*_*corrected*_ as *D*_*corr*._, $Score_{corrected}^{U}$ as $Score_{corr.}^{U}$, $Score_{corrected}^{null-info}$ as $Score_{corr.}^{null-info}$, $Score_{restriktor}^{null-info}$ as $Score_{restr.}^{null-info}$, $Score_{corrected}^{info}$ as $Score_{corr.}^{info}$, ${\bar{F}}_{corrected}$ as ${\bar{F}}_{corr.}$, *F*_*corrected*_ as *F*_*corr*._, *t*_*one*−*sided*_ as *t*_*one*−*s*._ and *t*_*two*−*sided*_ as *t*_*two*−*s*._. Bold values are above 0.06 and underlined values are below 0.04.

**Table 4:** Type I error rates when using ***R***_2_ and applying the test statistics as outlined in the referenced books.

				$Wald_{corr.}^{info}$						
***n***	***LRT*_*corr*._**	***LRT*_*restr*._**	$Wald_{naive}^{info}$	***Wald* ^*VCOV*^**	$Score_{corr.}^{U}$	$Score_{corr.}^{null-info}$	$Score_{restr.}^{null-info}$	$Score_{corr.}^{info}$	${\bar{F}}_{corr.}$	***F*_*corr*._**
				***D*_*corr*._**				***Score* ^*VCOV*^**	${\bar{F}}_{restr.}$	
10000	0.052	0.052	0.052	0.052	0.049	0.052	0.052	0.052	0.052	0.049
2000	0.048	0.048	0.050	0.048	0.052	0.048	0.048	0.048	0.048	0.046
1000	0.051	0.051	0.051	0.051	0.052	0.051	0.047	0.047	0.051	0.058
500	0.059	0.059	**0.062**	0.060	0.059	0.059	0.057	0.057	0.059	0.048
100	0.057	0.056	**0.070**	**0.061**	**0.078**	0.055	0.053	0.051	0.056	0.058
50	0.051	0.046	**0.090**	0.060	**0.099**	0.044	0.039	0.035	0.048	0.055
25	**0.068**	0.055	**0.135**	**0.083**	**0.119**	0.052	0.027	0.010	**0.064**	0.055
10	**0.069**	0.011	**0.416**	**0.163**	**0.334**	0.024	0.001	0.000	0.054	**0.061**

The test statistics are abbreviated as follows: *LRT*_*corrected*_ as *LRT*_*corr*._, *LRT*_*restriktor*_ as *LRT*_*restr*._, $Wald_{corrected}^{info}$ as $Wald_{corr.}^{info}$, *D*_*corrected*_ as *D*_*corr*._, $Score_{corrected}^{U}$ as $Score_{corr.}^{U}$, $Score_{corrected}^{null-info}$ as $Score_{corr.}^{null-info}$, $Score_{restriktor}^{null-info}$ as $Score_{restr.}^{null-info}$, $Score_{corrected}^{info}$ as $Score_{corr.}^{info}$, ${\bar{F}}_{corrected}$ as ${\bar{F}}_{corr.}$, *F*_*corrected*_ as *F*_*corr*._. Bold values are above 0.06 and underlined values are below 0.04.

We can observe that when using ***R***_1_ (see Table 3), that is when testing a hypothesis concerning only one regression parameter, type I error rates are identical between *F* and *t*_*two*−*sided*_ as well as between $\bar{F}$ and *t*_*one*−*sided*_, showing the link between classical null hypothesis testing and informative hypothesis testing. When using ***R***_2_ (see Table 4), that is when testing a hypothesis concerning multiple regression parameters, problems with type I error rates seem to occur earlier as compared to when using ***R***_1_. More specifically, problematic type I error rates occur as early as with *n* = 500 or *n* = 100 when using ***R***_2_, but only start occurring with *n* = 50 or *n* = 25 when using ***R***_1_. Apart from that, $Score_{corrected}^{U}$ and $Wald_{naive}^{info}$ show the highest type I error rates for both ***R*** matrices, whereas $\bar{F}$ and ${\bar{F}}_{restriktor}$ show the most appropriate type I error rates for both ***R*** matrices. This is because the $\bar{F}$-distribution is more precise in small sample sizes as compared to the ${\bar{\chi }}^{2}$-distribution.

When using the $\bar{F}$-distribution instead of the ${\bar{\chi }}^{2}$-distribution when calculating the *p*-value for all test statistics, type I error rates are closer to the nominal level when sample sizes get smaller. This can be seen in Tables 5, 6 where a selection of test statistics are shown.

**Table 5:** Type I error rates when using ***R***_1_, ${Ŝ}_{corrected}^{2}$ (or ${\tilde{S}}_{corrected}^{2},{\bar{S}}_{corrected}^{2}$) and the $\bar{F}$-distribution for calculating the *p*-value.

***n***	***LRT*_*corr*._**	$Wald_{corr.}^{info}$	$Score_{corr.}^{U}$
		***D*_*corr*._**	
10000	0.047	0.047	0.047
2000	0.054	0.054	0.057
1000	0.057	0.057	0.058
500	0.055	0.055	0.053
100	0.052	0.049	0.052
50	0.055	0.049	**0.065**
25	**0.067**	0.057	**0.080**
10	**0.084**	0.054	**0.126**

The test statistics are abbreviated as follows: *LRT*_*corrected*_ as *LRT*_*corr*._, $Wald_{corrected}^{info}$ as $Wald_{corr.}^{info}$, *D*_*corrected*_ as *D*_*corr*._ and $Score_{corrected}^{U}$ as $Score_{corr.}^{U}$. Bold values are above 0.06 and underlined values are below 0.04.

**Table 6:** Type I error rates when using ***R***_2_, ${Ŝ}_{corrected}^{2}$ (or ${\tilde{S}}_{corrected}^{2},{\bar{S}}_{corrected}^{2}$) and the $\bar{F}$-distribution for calculating the *p*-value.

***n***	***LRT*_*corr*._**	$Wald_{corr.}^{info}$	$Score_{corr.}^{U}$
		***D*_*corr*._**	
10000	0.052	0.052	0.049
2000	0.048	0.048	0.051
1000	0.050	0.051	0.051
500	0.059	0.059	0.058
100	0.055	0.056	**0.072**
50	0.043	0.048	**0.085**
25	0.042	**0.064**	**0.097**
10	0.006	0.054	**0.161**

The test statistics are abbreviated as follows: *LRT*_*corrected*_ as *LRT*_*corr*._, $Wald_{corrected}^{info}$ as $Wald_{corr.}^{info}$, *D*_*corrected*_ as *D*_*corr*._ and $Score_{corrected}^{U}$ as $Score_{corr.}^{U}$. Bold values are above 0.06 and underlined values are below 0.04.

Furthermore, it can be observed that when using ***R***_1_, type I error rates increase when using *LRT*_*corrected*_ and $Score_{corrected}^{U}$ and *n* = 10 in contrast to *n* = 25. The same can only be observed for $Score_{corrected}^{U}$ when using ***R***_2_, but not for *LRT*_*corrected*_, where the type I error rate decreases quite substantially instead. More results can be found in “Data Sheet 4” in the Supplementary materials.

### Type II results

**Figure 1:**
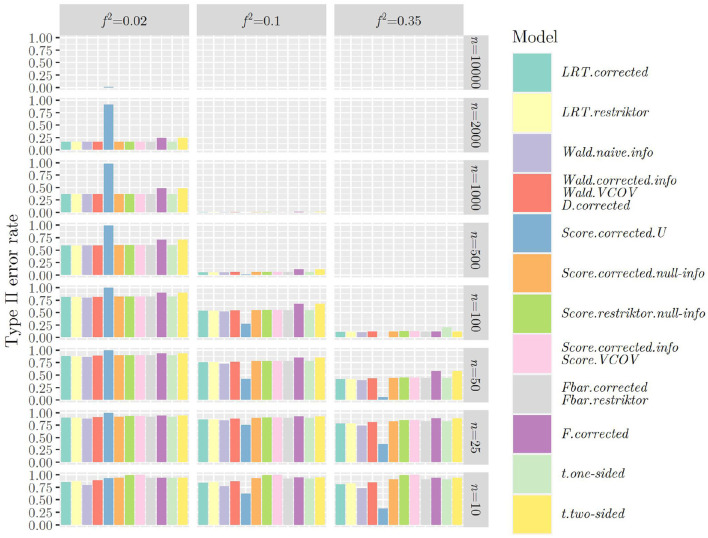
Type II error rates when using ***R***_1_ and applying the test statistics as outlined in the referenced books.

**Figure 2:**
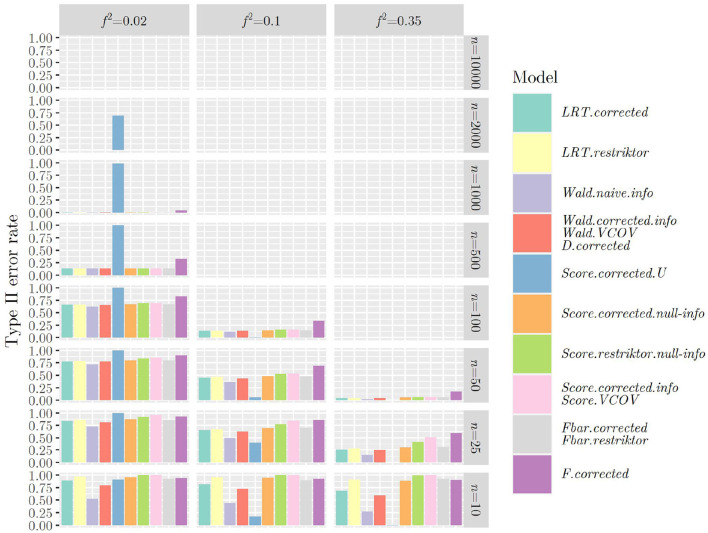
Type II error rates when using ***R***_2_ and applying the test statistics as outlined in the referenced books.

Figures 1, 2 show the type II error rates when applying the test statistics as in the referenced books.

Once more, we can observe that when using ***R***_1_ (see Figure 1), that is when testing a hypothesis concerning only one regression parameter, type II error rates are identical between *F* and *t*_*two*−*sided*_ as well as between $\bar{F}$ and *t*_*one*−*sided*_, showing the link between classical null hypothesis testing and informative hypothesis testing. When using ***R***_2_ (see Figure 2), that is when testing a hypothesis concerning multiple regression parameters, problems with type II error rates seem to occur later (in terms of sample size) as compared to when using ***R***_1_. This was the other way around regarding the type I error rate and it demonstrates the nature of the relationship between type I and type II error rates: If one goes down, the other one goes up and vice versa.

**Figure 3:**
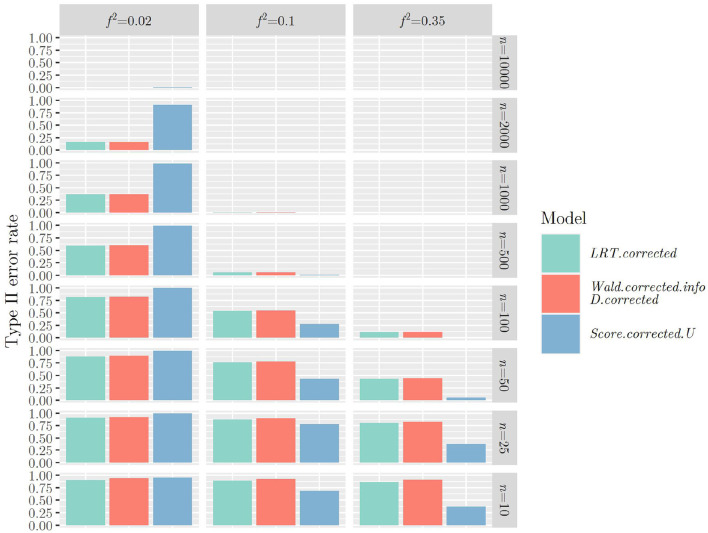
Type II error rates when using ***R***_1_, ${Ŝ}_{corrected}^{2}$ (or ${\tilde{S}}_{corrected}^{2},{\bar{S}}_{corrected}^{2}$) and the $\bar{F}$-distribution for calculating the *p*-value.

**Figure 4:**
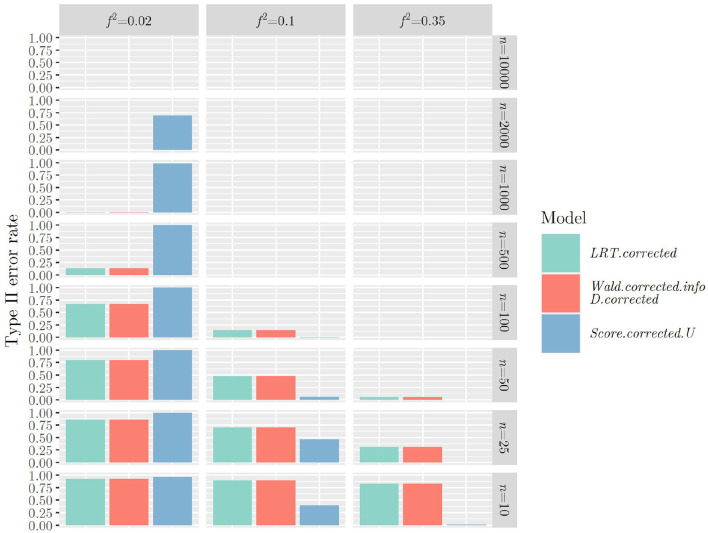
Type II error rates when using ***R***_2_, ${Ŝ}_{corrected}^{2}$ (or ${\tilde{S}}_{corrected}^{2},{\bar{S}}_{corrected}^{2}$) and the $\bar{F}$-distribution for calculating the *p*-value.

The same mechanism can be observed when using the $\bar{F}$-distribution instead of the ${\bar{\chi }}^{2}$-distribution when calculating the *p*-value for all test statistics (Figures 3, 4): Type II error rates are increased in small sample sizes, since type I error rates had improved, that is, decreased. Again, further results can be found in “Data Sheet 5” in the Supplementary materials.

## Discussion

In this paper, we gave an overview of a large number of different informative test statistics, including their different versions. Furthermore, we clarified how those test statistics perform in terms of type I and type II error rates under different conditions by means of simulation studies in the context of linear regression. We considered varying sample and effect sizes as well as two different constraint matrices, where one specified a hypothesis about one parameter and the other one specified a hypothesis about multiple parameters. Moreover, we considered the naive and corrected mean squared errors of the unconstrained, inequality and equality constrained models as part of the test statistics as well as the ${\bar{\chi }}^{2}$- and $\bar{F}$-distribution to calculate the *p*-values.

Based on our findings, the following recommendations can be made. Considering the time it takes to compute the informative test statistics, both the Wald and the $\bar{F}$-test versions are favorable, since they only need fitting of the inequality constrained model to obtain $\tilde{\beta }$ and $\tilde{I}$_1_. Even if we do not use $\tilde{I}$_1_ but use $\hat{I}$_1_ instead, the increase in time is small in the context of linear regression. The Score test and the LRT versions are less favorable, since they require fitting both the inequality constrained as well as the equality constrained model to obtain $\tilde{\beta }$ and $\bar{\beta }$, as well as the respective unit information matrices or log-likelihoods.

The *D*-statistic versions only require fitting the unconstrained model to obtain $\hat{\beta }$. However, we then additionally need to compute the two functions $d\left(\bar{\beta }\right)$ and $d\left(\tilde{\beta }\right)$, which is as time-consuming as fitting the inequality constrained model. Thus, there is no advantage of using the *D*-statistic versions over the Wald and the $\bar{F}$-test versions in the context of linear regression. However, if the regression model was non-linear, computing the two functions would be significantly less computationally expensive than fitting the inequality constrained model.

Moreover, we recommend using the corrected mean squared error versions in the test statistics as well as using the $\bar{F}$-distribution for calculating the *p*-values, if sample sizes are small. This seems to keep type I error rates closer to the nominal level compared to using the naive mean squared error versions and using the ${\bar{\chi }}^{2}$-distribution for calculating the *p*-value. An additional interesting finding was that the relationship between LRT, Wald and Score test values that has been found in the unconstrained context also holds in the constrained context. That is, Wald test values are always slightly larger than LRT values, which in turn are always slightly larger than Score test values.

The limitations of our simulation studies include the following aspects. We treated all variables as manifest, even though variables of interest in the social and behavioral sciences are often latent in nature. Furthermore, we solely generated normal data despite the fact that violations against the normality assumption occur regularly. Moreover, we used orthogonal predictors without interactions albeit this is rarely the case in the social and behavioral sciences. And lastly, we only included the regular versions of the standard errors and the variance-covariance matrix. Future research should thus repeat the simulation studies in the context of Structural Equation Modeling (SEM) to take into account latent variables. Furthermore, the impact of non-normal data as well as correlated predictors with interactions and using the robust versions of the standard errors and the variance-covariance matrix should be examined. It may be that under these conditions, type I and type II error rates deviate from the results presented in this paper. Moreover, the properties of informative test statistics, especially concerning the *D*-statistic, should also be investigated in the context of non-linear models.

Finally, research in the social and behavioral sciences is often not only interested in inference concerning regression coefficients, but also regarding effects of interest. These effects may be average or conditional treatment effects, which are defined as a linear or non-linear combination of regression coefficients. The EffectLiteR approach (Mayer et al., 2016) provides a framework and R package for the estimation of average and conditional effects of a discrete treatment variable on a continuous outcome variable, conditioning on categorical and continuous covariates. Keck et al. (2021) already demonstrated how to integrate informative hypothesis testing into the EffectLiteR framework in the context of linear regression. The present paper provides interested readers who want to apply informative hypothesis testing concerning regression coefficients or effects of interest with practical information regarding test statistics as well as type I and type II error rates.

## Data availability statement

The raw data supporting the conclusions of this article will be made available by the authors, without undue reservation.

## Author contributions

CK and YR contributed to conception and design of the study. CK performed the statistical analysis and wrote the first draft of the manuscript. CK, YR, and AM wrote sections of the manuscript. All authors contributed to manuscript revision, read, and approved the submitted version.

## Funding

This work has been supported by the Research Foundation Flanders (FWO, Grant No. G002819N to YR and AM).

## Conflict of interest

The authors declare that the research was conducted in the absence of any commercial or financial relationships that could be construed as a potential conflict of interest.

## Publisher's note

All claims expressed in this article are solely those of the authors and do not necessarily represent those of their affiliated organizations, or those of the publisher, the editors and the reviewers. Any product that may be evaluated in this article, or claim that may be made by its manufacturer, is not guaranteed or endorsed by the publisher.
