# Supplementary material for: Overview and evaluation of various frequentist test statistics using constrained statistical inference in the context of linear regression
Source: Front Psychol. 2022 Oct 14;13:899165. doi: 10.3389/fpsyg.2022.899165 (PMC9614349; doi:10.3389/fpsyg.2022.899165)
Supplement: Supplementary file 1 [file Data_Sheet_1.PDF]

Quadratic program in the D-statistic

The quadratic function that we try to minimize with respect to $\boldsymbol{\beta}$ as part of the D-statistic is:

$$\begin{aligned} f(\boldsymbol{\beta}) &= (\hat{\boldsymbol{\beta}} - \boldsymbol{\beta})'\boldsymbol{W}(\hat{\boldsymbol{\beta}} - \boldsymbol{\beta}) \\ &= \hat{\boldsymbol{\beta}}'\boldsymbol{W}\hat{\boldsymbol{\beta}} - \hat{\boldsymbol{\beta}}'\boldsymbol{W}\boldsymbol{\beta} - \boldsymbol{\beta}'\boldsymbol{W}\hat{\boldsymbol{\beta}} + \boldsymbol{\beta}'\boldsymbol{W}\boldsymbol{\beta}. \end{aligned}$$

$\boldsymbol{W}$ and $\hat{\boldsymbol{\beta}}$ are treated as fixed constants. Since the first term $\hat{\boldsymbol{\beta}}'\boldsymbol{W}\hat{\boldsymbol{\beta}}$ does not depend on $\boldsymbol{\beta}$, this equals:

$$= -2\hat{\boldsymbol{\beta}}'\boldsymbol{W}\boldsymbol{\beta} + \boldsymbol{\beta}'\boldsymbol{W}\boldsymbol{\beta}.$$

If we divide by 2, we obtain the form needed to use `solve.QP()`:

$$= -\hat{\boldsymbol{\beta}}'\boldsymbol{W}\boldsymbol{\beta} + \frac{1}{2} \times \boldsymbol{\beta}'\boldsymbol{W}\boldsymbol{\beta}.$$
