# Supplementary material for: Overview and evaluation of various frequentist test statistics using constrained statistical inference in the context of linear regression
Source: Front Psychol. 2022 Oct 14;13:899165. doi: 10.3389/fpsyg.2022.899165 (PMC9614349; doi:10.3389/fpsyg.2022.899165)
Supplement: Supplementary file 2 [file Data_Sheet_2.PDF]

Type I error rates: Further results

Table 1

Type I error rates when using $\boldsymbol{R}_1$, S^2_{naive} (or $\tilde{S}^2_{naive}, \bar{S}^2_{naive}$) and the $\bar{\chi}^2$-distribution for calculating the p-value. Bold values are above .06 and and underlined values are below .04.

n	LRT_{naive}	$Wald^{info}_{naive}$ D_{naive}	$Score^{U}_{naive}$
10 000	0.047	0.047	0.047
2000	0.054	0.054	0.058
1000	0.058	0.058	0.059
500	0.058	0.058	0.055
100	0.056	0.056	**0.067**
50	0.059	0.060	**0.094**
25	**0.086**	**0.092**	**0.143**
10	**0.173**	**0.186**	**0.326**

Table 2

Type I error rates when using $\boldsymbol{R}_2$, S^2_{naive} (or $\tilde{S}^2_{naive}, \bar{S}^2_{naive}$) and the $\bar{\chi}^2$-distribution for calculating the p-value. Bold values are above .06 and and underlined values are below .04.

n	LRT_{naive}	$Wald^{info}_{naive}$ D_{naive}	$Score^{U}_{naive}$
10 000	0.052	0.052	0.049
2000	0.048	0.050	0.052
1000	0.051	0.051	0.053
500	0.059	**0.062**	**0.061**
100	0.059	**0.070**	**0.093**
50	**0.061**	**0.090**	**0.128**
25	**0.084**	**0.135**	**0.211**
10	**0.165**	**0.416**	**0.542**

Table 3

Type I error rates when using $\boldsymbol{R}_1$, S^2_{naive} (or $\tilde{S}^2_{naive}, \bar{S}^2_{naive}$) and the $\bar{F}$-distribution for calculating the p-value. Bold values are above .06 *and and underlined values are below* .04.

n	LRT_{naive}	$Wald^{info}_{naive}$ D_{naive}	$Score^{U}_{naive}$
10 000	0.047	0.047	0.047
2000	0.054	0.054	0.058
1000	0.057	0.058	0.059
500	0.058	0.058	0.055
100	0.054	0.056	**0.063**
50	0.058	0.059	**0.089**
25	**0.074**	**0.078**	**0.137**
10	**0.112**	**0.130**	**0.289**

Table 4

Type I error rates when using $\boldsymbol{R}_2$, S^2_{naive} (or $\tilde{S}^2_{naive}, \bar{S}^2_{naive}$) and the $\bar{F}$-distribution for calculating the p-value. Bold values are above .06 *and and underlined values are below* .04.

n	LRT_{naive}	$Wald^{info}_{naive}$ D_{naive}	$Score^{U}_{naive}$
10 000	0.052	0.052	0.049
2000	0.048	0.048	0.052
1000	0.051	0.051	0.052
500	0.059	0.060	**0.061**
100	0.056	**0.062**	**0.087**
50	0.046	**0.069**	**0.120**
25	0.055	**0.102**	**0.171**
10	0.011	**0.173**	**0.345**
