# Supplementary material for: Overview and evaluation of various frequentist test statistics using constrained statistical inference in the context of linear regression
Source: Front Psychol. 2022 Oct 14;13:899165. doi: 10.3389/fpsyg.2022.899165 (PMC9614349; doi:10.3389/fpsyg.2022.899165)
Supplement: Supplementary file 3 [file Data_Sheet_3.PDF]

Type II error rates: Further results

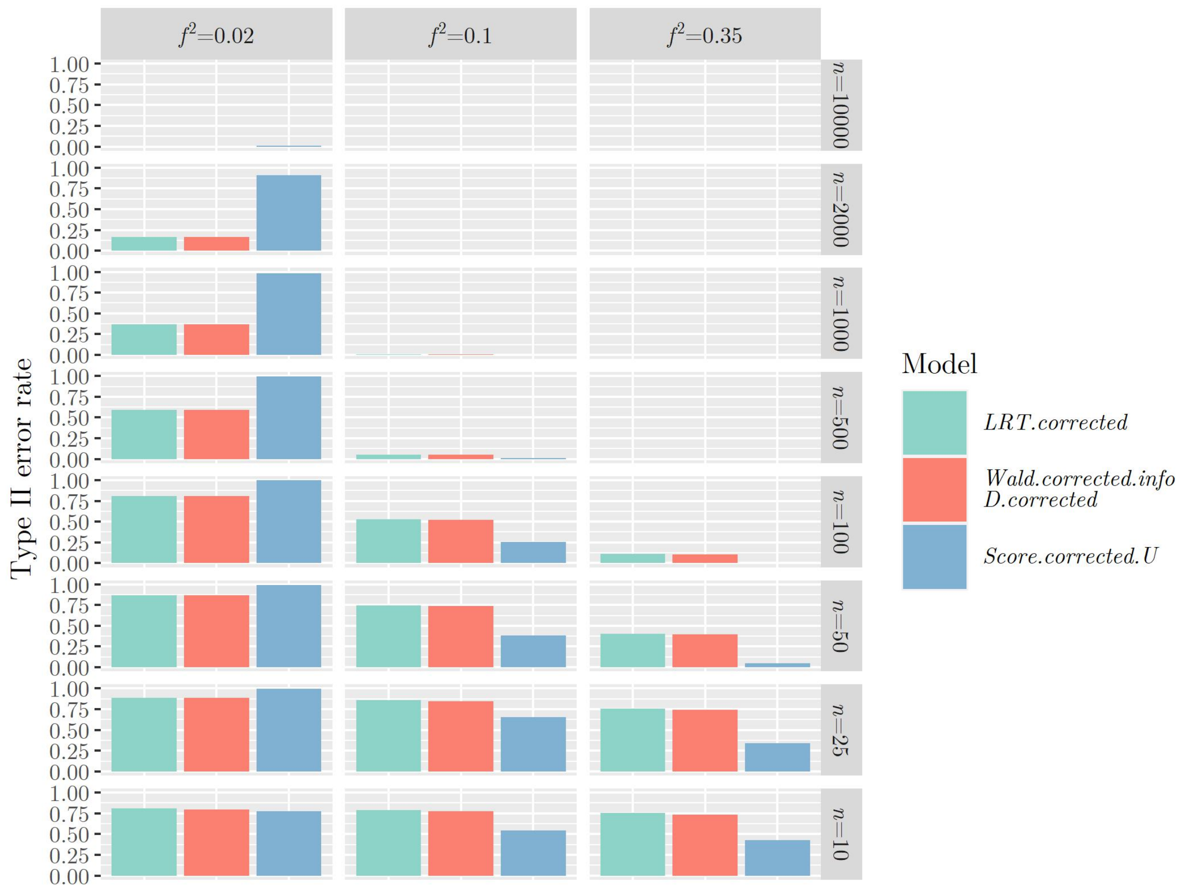

Figure 1. Type II error rates when using $\boldsymbol{R}_1$, $\hat{S}^2_{naive}$ (or $\tilde{S}^2_{naive}$, $\bar{S}^2_{naive}$) and the $\bar{\chi}^2$-distribution for calculating the p-value.

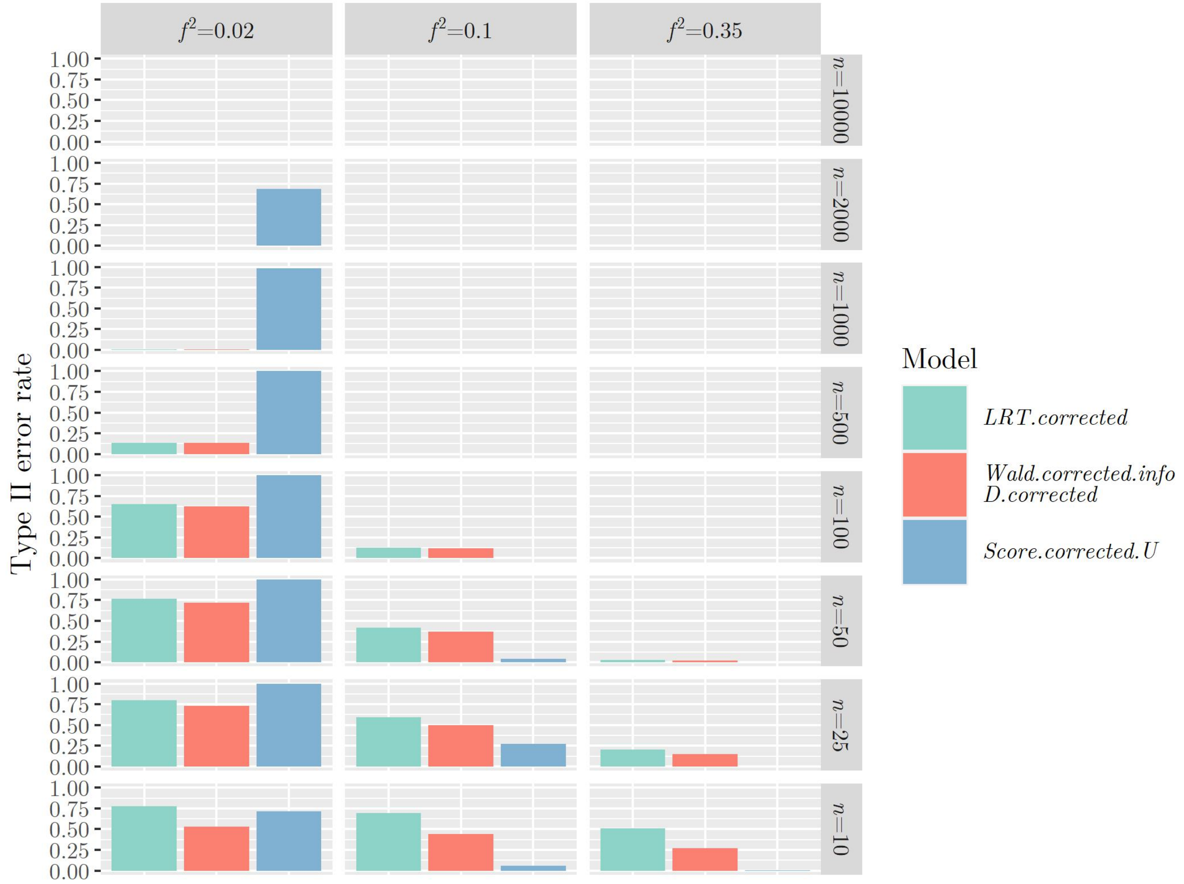

Figure 2. Type II error rates when using $\boldsymbol{R}_2$, $\hat{S}^2_{naive}$ (or $\tilde{S}^2_{naive}$, $\bar{S}^2_{naive}$) and the $\bar{\chi}^2$-distribution for calculating the p-value.

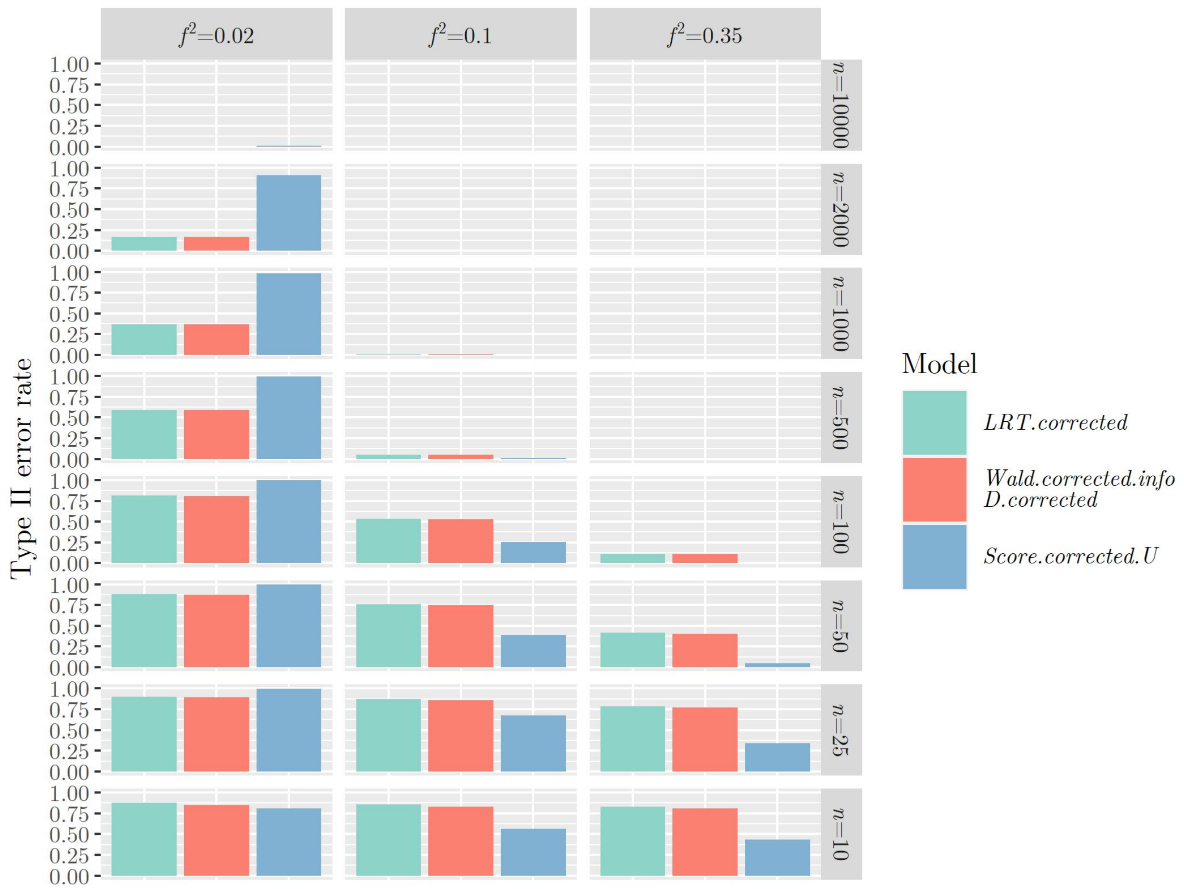

Figure 3. Type II error rates when using $\boldsymbol{R}_1$, $\hat{S}^2_{naive}$ (or $\tilde{S}^2_{naive}, \bar{S}^2_{naive}$) and the $\bar{F}$-distribution for calculating the p-value.

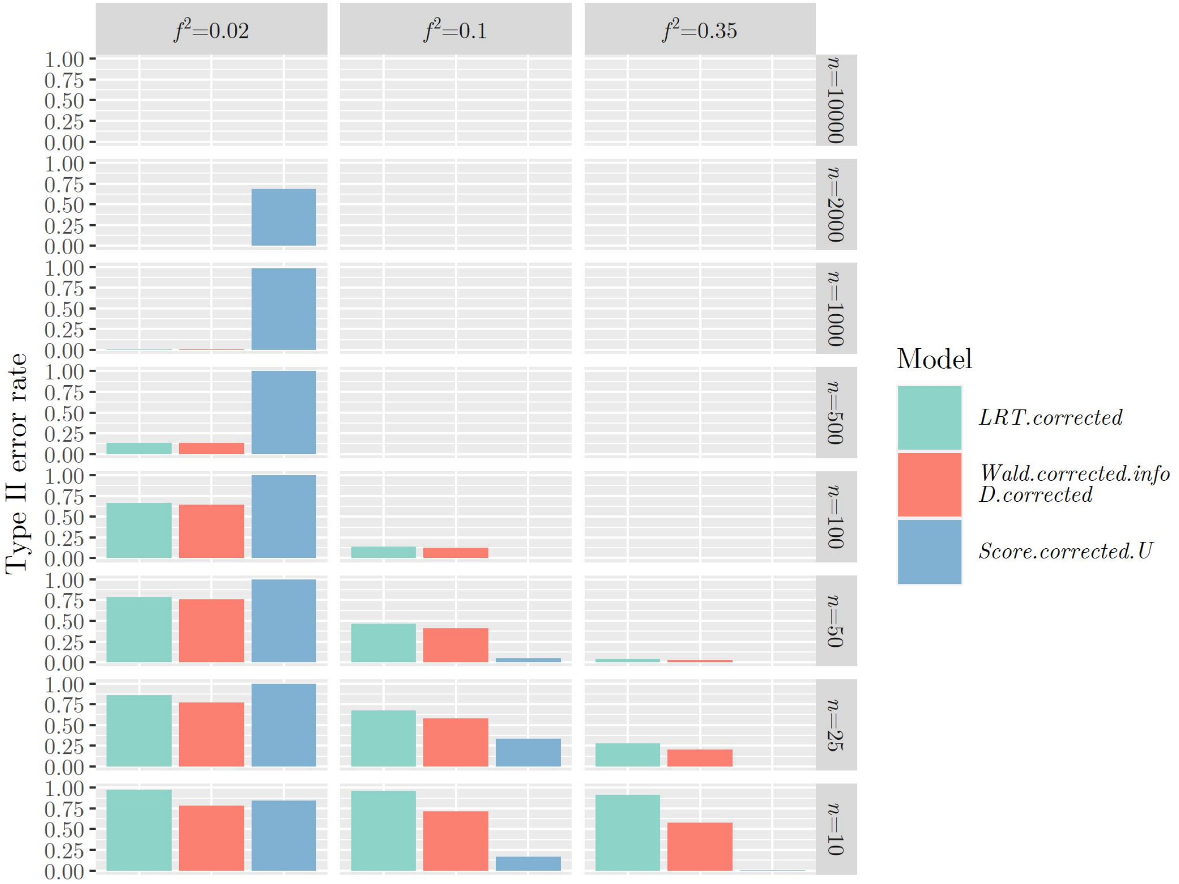

Figure 4. Type II error rates when using $\boldsymbol{R}_2$, $\hat{S}^2_{naive}$ (or $\tilde{S}^2_{naive}, \bar{S}^2_{naive}$) and the $\bar{F}$-distribution for calculating the p-value.
