# Supplementary material for: Overview and evaluation of various frequentist test statistics using constrained statistical inference in the context of linear regression
Source: Front Psychol. 2022 Oct 14;13:899165. doi: 10.3389/fpsyg.2022.899165 (PMC9614349; doi:10.3389/fpsyg.2022.899165)
Supplement: Supplementary file 4 [file Data_Sheet_4.PDF]

Test statistics R code

```
library(quadprog)
library(restriktor)

# the test statistics mentioned in this script can be found in
# Allen, M. P. (1997). Understanding regression analysis. Boston, MA: Springer.
# Buse, A. (1982). The likelihood ratio, wald and langrange multiplier tests: An
# expository note. The American Statistician, 36(3), 153-157.
# Seber, G. A. F., & Lee, A. J. (2012). Linear regression analysis. Hoboken, NJ:
# Wiley.
# Silvapulle, M. J., & Sen, P. K. (2005). Constrained statistical inference:
# Order, inequality, and shape restrictions. Hoboken, NJ: Wiley.
# Silvapulle, M. J., & Silvapulle, P. (1995). A score test against one-sided
# alternatives. Journal of the American Statistical Association, 90(429),
# 342-349.


# function to compute the p-value using the chibarsquare-distribution
# Silvapulle & Sen (2005), p. 86
pchisqbar <- function(x, df, wt.bar) {
  if (x <= 0) {
    return(0)
  }
  zed <- df == 0
  cdf <- ifelse(any(zed), wt.bar[zed], 0)
  cdf <- cdf + sum(pchisq(x, df[!zed]) * wt.bar[!zed])
  return(cdf)
}

# function to compute the p-value using the fbar-distribution
# Silvapulle & Sen (2005), p. 99
pfbar <- function (x, df1, df2, wt.bar) {
  if (x <= 0) {
    return(0)
  }
  zed <- df1 == 0
  cdf <- ifelse(any(zed), wt.bar[zed], 0)
  cdf <- cdf + sum(pf(x/df1[!zed], df1[!zed], df2) * wt.bar[!zed])
  return(cdf)
}


# generate data

```

```
# number of regression coefficients (without intercept)
p <- 5
# number of observations
N <- 1000

# R matrices
R1 <- rbind(c(0, 1, 0, 0, 0, 0))
R2 <- rbind(c(0, 1, 0, 0, 0, 0),
            c(0, 0, 1, 0, 0, 0))


# create design matrix
set.seed(2345)
X.fixed <- MASS:::mvrnorm(N, mu = rep(0, p), Sigma = diag(p), empirical = FALSE)

# create random y
beta.pop <- c(10, -0.1, 0.2, 3, 4, 5)
y <- cbind(1, X.fixed) %*% beta.pop + rnorm(N, sd = 0.5)
d <- data.frame(y = y, X = X.fixed)


#### analysis using R1 ####

# fit unrestricted linear regression model and obtain relevant quantities
fit <- lm(y ~ X.1 + X.2 + X.3 + X.4 + X.5, data = d)
beta.hat <- coef(fit)
VCOV <- vcov(fit)
X <- model.matrix(fit)[,]
y <- d$y
W <- 1/N * crossprod(X)
k <- ncol(X)
h <- nrow(R1)
resid.hat <- drop(y - X %*% beta.hat)
s2.hat.corr <- sum(resid.hat^2)/(N-k)
s2.hat.naive <- sum(resid.hat^2)/N
I.hat.corr <- 1/(N*s2.hat.corr) * crossprod(X)
I.hat.naive <- 1/(N*s2.hat.naive) * crossprod(X)


# fit constrained models with restriktor and obtain weights
fit.restr <- restriktor(fit, constraints = R1, se = "none")
wt.bar <- fit.restr$wt.bar

# compute quadratic program with inequality constraints
```

```
# and obtain relevant quantities
fit.ineq <- solve.QP(Dmat = crossprod(X), dvec = crossprod(y,X), Amat = t(R1),
                     bvec = rep(0, h), meq = 0L)
beta.tilde <- fit.ineq $solution
resid.tilde <- drop(y - X %*% beta.tilde)
s2.tilde.corr<- sum(resid.tilde ^2)/(N - k)
s2.tilde.naive <- sum(resid.tilde^2)/N
I.tilde.corr <- 1/(N*s2.tilde.corr) * crossprod(X)
I.tilde.naive <- 1/(N*s2.tilde.naive) * crossprod(X)

# compute quadratic program with equality constraints
# and obtain relevant quantities
fit.eq <- solve.QP(Dmat = crossprod(X), dvec = crossprod(y,X), Amat = t(R1),
                   bvec = rep(0, h), meq = nrow(R1))
beta.bar <- fit.eq$solution
resid.bar <- drop(y - X %*% beta.bar)
s2.bar.corr <- sum(resid.bar^2)/(N - (k - h))
s2.bar.naive <- sum(resid.bar^2)/N
I.bar.corr <- 1/(N*s2.bar.corr) * crossprod(X)
I.bar.naive <- 1/(N*s2.bar.naive) * crossprod(X)


# regular hypothesis testing

# Wald using corrected unit information matrix
# Buse (1982), p. 154
W.reg <- drop(N * t(R1 %*% beta.hat) %*% solve(R1 %*% solve(I.hat.corr) %*%
                                              t(R1)) %*% R1 %*% beta.hat)
1 - pchisq(W.reg, df = h)
1 - pf(W.reg, df1 = h, df2 = N-p-1) # considering the intercept

# LRT using corrected mean squared error terms
# Buse (1982), p. 153
logl.bar.corr <- sum(dnorm(resid.bar, sd = sqrt(s2.bar.corr), log = TRUE))
logl.hat.corr <- sum(dnorm(resid.hat, sd = sqrt(s2.hat.corr), log = TRUE))
LRT.reg <- -2 * (logl.bar.corr - logl.hat.corr)
1 - pchisq(LRT.reg, df = h)
1 - pf(LRT.reg, df1 = h, df2 = N-p-1)

# Score using corrected unit information matrix and corrected mean squared
# error terms
# Buse (1982), p. 155
S.bar.corr   <- 1/s2.bar.corr * (t(X) %*% y - t(X) %*% X %*% beta.bar)
Score.reg <- drop(1/N * t(S.bar.corr) %*% solve(I.bar.corr) %*% S.bar.corr)
```

```r
1 - pchisq(Score.reg, df = h)
1 - pf(Score.reg, df1 = h, df2 = N-p-1)

# F using corrected unit information matrix
# Seber & Lee (2012), p. 100
F.reg <- drop(N/h * t(R1 %*% beta.hat) %*%
                solve(R1 %*% solve(I.hat.corr) %*% t(R1)) %*% R1 %*% beta.hat)
1 - pf(F.reg, df1 = h, df2 = N-p-1)

# relationship between F and W in regular null hypothesis testing
F.reg*h == W.reg

# t using corrected mean squared error
# Allen (1997), p. 67
se.beta.hat <- sqrt(s2.hat.corr * diag(solve(t(X)%*%X)))
t <- beta.hat/se.beta.hat

# one-sided p-value
if(t[2] < 0) {
  pt(abs(t[2]), N-p-1)
} else {
  1 - pt(abs(t[2]), N-p-1)
}

# two-sided p-value
2 * (1 - pt(abs(t[2]), N-p-1))

# relationship t and F as long as h=1
round(t[2]^2) == round(F.reg)


# informative hypothesis testing using restriktor

# Fbar
Fbar.restr <- iht(fit.restr, test = "f", type = "a")$Ts
iht(fit.restr, test = "f", type = "a")$pvalue[1]

# LRT.corrected
LRT.restr <- iht(fit.restr, test = "lrt", type = "a")$Ts
iht(fit.restr, test = "lrt", type = "a")$pvalue[1]

# Score.null-info.corrected
Score.restr <- iht(fit.restr, test = "score", type = "a")$Ts
iht(fit.restr, test = "score", type = "a")$pvalue[1]
```

```


# manual informative hypothesis testing

# LRT using corrected mean squared error terms
# Silvapulle & Sen (2005), p. 157
logl.tilde.corr <- sum(dnorm(resid.tilde, sd = sqrt(s2.tilde.corr), log = TRUE))
LRT.corr <- -2 * (logl.bar.corr - logl.tilde.corr)
1 - pchisqbar(LRT.corr, df = 0:h, wt.bar = wt.bar)
1 - pfbar(LRT.corr, df1 = 0:h, df2 = nrow(X)-ncol(X), wt.bar = wt.bar)

# LRT using naive mean squared error terms
logl.bar.naive <- sum(dnorm(resid.bar, sd = sqrt(s2.bar.naive), log = TRUE))
logl.tilde.naive <- sum(dnorm(resid.tilde, sd = sqrt(s2.tilde.naive),
                              log = TRUE))
LRT.naive <- -2 * (logl.bar.naive - logl.tilde.naive)
1 - pchisqbar(LRT.naive, df = 0:h, wt.bar = wt.bar)
1 - pfbar(LRT.naive, df1 = 0:h, df2 = nrow(X)-ncol(X), wt.bar = wt.bar)

# W.info.corr using corrected mean squared error
# Silvapulle & Sen (2005), p. 154
W.info.corr <- (N/s2.hat.corr) * t(R1 %*% beta.tilde) %*%
  solve(R1 %*% solve(W) %*% t(R1)) %*% R1 %*% beta.tilde
1 - pchisqbar(W.info.corr, df = 0:h, wt.bar = wt.bar)
1 - pfbar(W.info.corr, df1 = 0:h, df2 = nrow(X)-ncol(X), wt.bar = wt.bar)

# W.info.naive using naive mean squared error
W.info.naive <- (N/s2.hat.naive) * t(R1 %*% beta.tilde) %*%
  solve(R1 %*% solve(W) %*% t(R1)) %*% R1 %*% beta.tilde
1 - pchisqbar(W.info.naive, df = 0:h, wt.bar = wt.bar)
1 - pfbar(W.info.naive, df1 = 0:h, df2 = nrow(X)-ncol(X), wt.bar = wt.bar)

# W.VCOV
W.VCOV <- t(R1 %*% beta.tilde) %*%
  solve(R1 %*% VCOV %*% t(R1)) %*% R1 %*% beta.tilde
1 - pchisqbar(W.VCOV, df = 0:h, wt.bar = wt.bar)
1 - pfbar(W.VCOV, df1 = 0:h, df2 = nrow(X)-ncol(X), wt.bar = wt.bar)

# D.corr using corrected mean squared error
# Silvapulle& Sen (2005), p. 159
out.eq   <- solve.QP(Dmat = W, dvec = beta.hat %*% W, Amat = t(R1),
                     bvec = rep(0, h), meq = nrow(R1))
out.ineq <- solve.QP(Dmat = W, dvec = beta.hat %*% W, Amat = t(R1),
                     bvec = rep(0, h), meq = 0L)
```

```
D.corr <- (2 * N)/s2.hat.corr * (out.eq$value - out.ineq$value)
1 - pchisqbar(D.corr, df = 0:h, wt.bar = wt.bar)
1 - pfbar(D.corr, df1 = 0:h, df2 = nrow(X)-ncol(X), wt.bar = wt.bar)

# D.naive using naive mean squared error
D.naive <- (2 * N)/s2.hat.naive * (out.eq$value - out.ineq$value)
1 - pchisqbar(D.naive, df = 0:h, wt.bar = wt.bar)
1 - pfbar(D.naive, df1 = 0:h, df2 = nrow(X)-ncol(X), wt.bar = wt.bar)

# Fbar using corrected unit information matrix
# Silvapulle & Sen (2005), p. 29
Fbar.corr = N * t(R1 %*% beta.tilde) %*%
  solve(R1 %*% solve(I.hat.corr) %*% t(R1)) %*% R1 %*% beta.tilde
1 - pfbar(Fbar.corr, df1 = 0:h, df2 = nrow(X)-ncol(X), wt.bar = wt.bar)

# Fbar using naive unit information matrix
Fbar.naive = N * t(R1 %*% beta.tilde) %*%
  solve(R1 %*% solve(I.hat.naive) %*% t(R1)) %*% R1 %*% beta.tilde
1 - pfbar(Fbar.naive, df1 = 0:h, df2 = nrow(X)-ncol(X), wt.bar = wt.bar)

# relationship Fbar and Wald in informative hypothesis testing
round(Fbar.corr) == round(W.info.corr)

# Score.U.corr using corrected mean squared error terms
# Silvapulle & Sen (2005), p. 159
S.tilde.corr <- 1/s2.tilde.corr * (t(X) %*% y - t(X) %*% X %*% beta.tilde)
U.corr <- R1 %*% solve(W) %*% (S.tilde.corr - S.bar.corr)
Score.U.corr <- 1/(N*s2.hat.corr) * t(U.corr) %*%
  solve(R1 %*% solve(W) %*% t(R1)) %*% U.corr
1 - pchisqbar(Score.U.corr, df = 0:h, wt.bar = wt.bar)
1 - pfbar(Score.U.corr, df1 = 0:h, df2 = nrow(X)-ncol(X), wt.bar = wt.bar)

# Score.U.naive using naive mean squared error terms
S.tilde.naive <- 1/s2.tilde.naive * (t(X) %*% y - t(X) %*% X %*% beta.tilde)
S.bar.naive <- 1/s2.bar.naive * (t(X) %*% y - t(X) %*% X %*% beta.bar)
U.naive <- R1 %*% solve(W) %*% (S.tilde.naive - S.bar.naive)
Score.U.naive <- 1/(N*s2.hat.naive) * t(U.naive) %*%
  solve(R1 %*% solve(W) %*% t(R1)) %*% U.naive
1 - pchisqbar(Score.U.naive, df = 0:h, wt.bar = wt.bar)
1 - pfbar(Score.U.naive, df1 = 0:h, df2 = nrow(X)-ncol(X), wt.bar = wt.bar)

# Score.null-info.corr using corrected unit information matrix and corrected mean
# squared error terms
# Silvapulle & Silvapulle (1995), p. 342
```

```
Score.nullinfo.corr <- 1/N * c(S.bar.corr - S.tilde.corr) %*% solve(I.bar.corr) %*%
  (S.bar.corr - S.tilde.corr)
1 - pchisqbar(Score.nullinfo.corr, df = 0:h, wt.bar = wt.bar)
1 - pfbar(Score.nullinfo.corr, df1 = 0:h, df2 = nrow(X)-ncol(X), wt.bar = wt.bar)

# Score.null-info.naive using naive unit information matrix and naive mean squared
# error terms
Score.nullinfo.naive <- 1/N * c(S.bar.naive - S.tilde.naive) %*%
  solve(I.bar.naive) %*% (S.bar.naive - S.tilde.naive)
1 - pchisqbar(Score.nullinfo.naive, df = 0:h, wt.bar = wt.bar)
1 - pfbar(Score.nullinfo.naive, df1 = 0:h, df2 = nrow(X)-ncol(X), wt.bar = wt.bar)

# Score.info.corr using corrected unit information matrix
# Silvapulle & Sen (2005), p. 166
P.corr <- R1 %*% solve(I.hat.corr) %*% (S.tilde.corr-S.bar.corr)
Score.info.corr <- 1/N * t(P.corr) %*% solve(R1 %*% solve(I.hat.corr) %*%
                                             t(R1)) %*% P.corr
1 - pchisqbar(Score.info.corr, df = 0:h, wt.bar = wt.bar)
1 - pfbar(Score.info.corr, df1 = 0:h, df2 = nrow(X)-ncol(X), wt.bar = wt.bar)

# Score.info.naive using naive unit information matrix
P.naive <- R1 %*% solve(I.hat.naive) %*% (S.tilde.naive-S.bar.naive)
Score.info.naive <- 1/N * t(P.naive) %*% solve(R1 %*% solve(I.hat.naive) %*%
                                               t(R1)) %*% P.naive
1 - pchisqbar(Score.info.naive, df = 0:h, wt.bar = wt.bar)
1 - pfbar(Score.info.naive, df1 = 0:h, df2 = nrow(X)-ncol(X), wt.bar = wt.bar)

# Score.VCOV
V <- R1 %*% VCOV %*% (S.tilde.corr-S.bar.corr)
Score.VCOV <- t(V) %*% solve(R1 %*% VCOV %*% t(R1)) %*% V
1 - pchisqbar(Score.VCOV, df = 0:h, wt.bar = wt.bar)
1 - pfbar(Score.VCOV, df1 = 0:h, df2 = nrow(X)-ncol(X), wt.bar = wt.bar)


#### analysis using R2 ####

# fit unrestricted linear regression model and obtain relevant quantities
fit <- lm(y ~ X.1 + X.2 + X.3 + X.4 + X.5, data = d)
beta.hat <- coef(fit)
VCOV <- vcov(fit)
X <- model.matrix(fit)[,]
y <- d$y
W <- 1/N * crossprod(X)
k <- ncol(X)
```

```
h <- nrow(R2)
resid.hat <- drop(y - X %*% beta.hat)
s2.hat.corr <- sum(resid.hat^2)/(N-k)
s2.hat.naive <- sum(resid.hat^2)/N
I.hat.corr <- 1/(N*s2.hat.corr) * crossprod(X)
I.hat.naive <- 1/(N*s2.hat.naive) * crossprod(X)


# fit constrained models with restriktor and obtain weights
fit.restr <- restriktor(fit, constraints = R2, se = "none")
wt.bar <- fit.restr$wt.bar

# compute quadratic program with inequality constraints
# and obtain relevant quantities
fit.ineq <- solve.QP(Dmat = crossprod(X), dvec = crossprod(y,X), Amat = t(R2),
                     bvec = rep(0, h), meq = 0L)
beta.tilde <- fit.ineq $solution
resid.tilde <- drop(y - X %*% beta.tilde)
s2.tilde.corr<- sum(resid.tilde ^2)/(N - k)
s2.tilde.naive <- sum(resid.tilde^2)/N
I.tilde.corr <- 1/(N*s2.tilde.corr) * crossprod(X)
I.tilde.naive <- 1/(N*s2.tilde.naive) * crossprod(X)

# compute quadratic program with equality constraints
# and obtain relevant quantities
fit.eq <- solve.QP(Dmat = crossprod(X), dvec = crossprod(y,X), Amat = t(R2),
                   bvec = rep(0, h), meq = nrow(R2))
beta.bar <- fit.eq$solution
resid.bar <- drop(y - X %*% beta.bar)
s2.bar.corr <- sum(resid.bar^2)/(N - (k - h))
s2.bar.naive <- sum(resid.bar^2)/N
I.bar.corr <- 1/(N*s2.bar.corr) * crossprod(X)
I.bar.naive <- 1/(N*s2.bar.naive) * crossprod(X)


# regular hypothesis testing

# Wald using corrected unit information matrix
# Buse (1982), p. 154
W.reg <- drop(N * t(R2 %*% beta.hat) %*% solve(R2 %*% solve(I.hat.corr) %*%
                                               t(R2)) %*% R2 %*% beta.hat)
1 - pchisq(W.reg, df = h)
1 - pf(W.reg, df1 = h, df2 = N-p-1) # considering the intercept

```

```r
# LRT using corrected mean squared error terms
# Buse (1982), p. 153
logl.bar.corr <- sum(dnorm(resid.bar, sd = sqrt(s2.bar.corr), log = TRUE))
logl.hat.corr <- sum(dnorm(resid.hat, sd = sqrt(s2.hat.corr), log = TRUE))
LRT.reg <- -2 * (logl.bar.corr - logl.hat.corr)
1 - pchisq(LRT.reg, df = h)
1 - pf(LRT.reg, df1 = h, df2 = N-p-1)

# Score using corrected unit information matrix and corrected mean squared
# error terms
# Buse (1982), p. 155
S.bar.corr   <- 1/s2.bar.corr * (t(X) %*% y - t(X) %*% X %*% beta.bar)
Score.reg <- drop(1/N * t(S.bar.corr) %*% solve(I.bar.corr) %*% S.bar.corr)
1 - pchisq(Score.reg, df = h)
1 - pf(Score.reg, df1 = h, df2 = N-p-1)

# F using corrected unit information matrix
# Seber & Lee (2012), p. 100
F.reg <- drop(N/h * t(R2 %*% beta.hat) %*%
                solve(R2 %*% solve(I.hat.corr) %*% t(R2)) %*% R2 %*% beta.hat)
1 - pf(F.reg, df1 = h, df2 = N-p-1)

# relationship between F and W in regular null hypothesis testing
F.reg*h == W.reg


# informative hypothesis testing using restriktor

# Fbar
Fbar.restr <- iht(fit.restr, test = "f", type = "a")$Ts
iht(fit.restr, test = "f", type = "a")$pvalue[1]

# LRT.corrected
LRT.restr <- iht(fit.restr, test = "lrt", type = "a")$Ts
iht(fit.restr, test = "lrt", type = "a")$pvalue[1]

# Score.null-info.corrected
Score.restr <- iht(fit.restr, test = "score", type = "a")$Ts
iht(fit.restr, test = "score", type = "a")$pvalue[1]


# manual informative hypothesis testing

# LRT using corrected mean squared error terms
```

```r
# Silvapulle & Sen (2005), p. 157
logl.tilde.corr <- sum(dnorm(resid.tilde, sd = sqrt(s2.tilde.corr), log = TRUE))
LRT.corr <- -2 * (logl.bar.corr - logl.tilde.corr)
1 - pchisqbar(LRT.corr, df = 0:h, wt.bar = wt.bar)
1 - pfbar(LRT.corr, df1 = 0:h, df2 = nrow(X)-ncol(X), wt.bar = wt.bar)

# LRT using naive mean squared error terms
logl.bar.naive <- sum(dnorm(resid.bar, sd = sqrt(s2.bar.naive), log = TRUE))
logl.tilde.naive <- sum(dnorm(resid.tilde, sd = sqrt(s2.tilde.naive),
                              log = TRUE))
LRT.naive <- -2 * (logl.bar.naive - logl.tilde.naive)
1 - pchisqbar(LRT.naive, df = 0:h, wt.bar = wt.bar)
1 - pfbar(LRT.naive, df1 = 0:h, df2 = nrow(X)-ncol(X), wt.bar = wt.bar)

# W.info.corr using corrected mean squared error
# Silvapulle & Sen (2005), p. 154
W.info.corr <- (N/s2.hat.corr) * t(R2 %*% beta.tilde) %*%
  solve(R2 %*% solve(W) %*% t(R2)) %*% R2 %*% beta.tilde
1 - pchisqbar(W.info.corr, df = 0:h, wt.bar = wt.bar)
1 - pfbar(W.info.corr, df1 = 0:h, df2 = nrow(X)-ncol(X), wt.bar = wt.bar)

# W.info.naive using naive mean squared error
W.info.naive <- (N/s2.hat.naive) * t(R2 %*% beta.tilde) %*%
  solve(R2 %*% solve(W) %*% t(R2)) %*% R2 %*% beta.tilde
1 - pchisqbar(W.info.naive, df = 0:h, wt.bar = wt.bar)
1 - pfbar(W.info.naive, df1 = 0:h, df2 = nrow(X)-ncol(X), wt.bar = wt.bar)

# W.VCOV
W.VCOV <- t(R2 %*% beta.tilde) %*%
  solve(R2 %*% VCOV %*% t(R2)) %*% R2 %*% beta.tilde
1 - pchisqbar(W.VCOV, df = 0:h, wt.bar = wt.bar)
1 - pfbar(W.VCOV, df1 = 0:h, df2 = nrow(X)-ncol(X), wt.bar = wt.bar)

# D.corr using corrected mean squared error
# Silvapulle& Sen (2005), p. 159
out.eq   <- solve.QP(Dmat = W, dvec = beta.hat %*% W, Amat = t(R2),
                     bvec = rep(0, h), meq = nrow(R2))
out.ineq <- solve.QP(Dmat = W, dvec = beta.hat %*% W, Amat = t(R2),
                     bvec = rep(0, h), meq = 0L)
D.corr <- (2 * N)/s2.hat.corr * (out.eq$value - out.ineq$value)
1 - pchisqbar(D.corr, df = 0:h, wt.bar = wt.bar)
1 - pfbar(D.corr, df1 = 0:h, df2 = nrow(X)-ncol(X), wt.bar = wt.bar)

# D.naive using naive mean squared error
```

```
D.naive <- (2 * N)/s2.hat.naive * (out.eq$value - out.ineq$value)
1 - pchisqbar(D.naive, df = 0:h, wt.bar = wt.bar)
1 - pfbar(D.naive, df1 = 0:h, df2 = nrow(X)-ncol(X), wt.bar = wt.bar)

# Fbar using corrected unit information matrix
# Silvapulle & Sen (2005), p. 29
Fbar.corr = N * t(R2 %*% beta.tilde) %*%
  solve(R2 %*% solve(I.hat.corr) %*% t(R2)) %*% R2 %*% beta.tilde
1 - pfbar(Fbar.corr, df1 = 0:h, df2 = nrow(X)-ncol(X), wt.bar = wt.bar)

# Fbar using naive unit information matrix
Fbar.naive = N * t(R2 %*% beta.tilde) %*%
  solve(R2 %*% solve(I.hat.naive) %*% t(R2)) %*% R2 %*% beta.tilde
1 - pfbar(Fbar.naive, df1 = 0:h, df2 = nrow(X)-ncol(X), wt.bar = wt.bar)

# relationship Fbar and Wald in informative hypothesis testing
round(Fbar.corr) == round(W.info.corr)

# Score.U.corr using corrected mean squared error terms
# Silvapulle & Sen (2005), p. 159
S.tilde.corr <- 1/s2.tilde.corr * (t(X) %*% y - t(X) %*% X %*% beta.tilde)
U.corr <- R2 %*% solve(W) %*% (S.tilde.corr - S.bar.corr)
Score.U.corr <- 1/(N*s2.hat.corr) * t(U.corr) %*%
  solve(R2 %*% solve(W) %*% t(R2)) %*% U.corr
1 - pchisqbar(Score.U.corr, df = 0:h, wt.bar = wt.bar)
1 - pfbar(Score.U.corr, df1 = 0:h, df2 = nrow(X)-ncol(X), wt.bar = wt.bar)

# Score.U.naive using naive mean squared error terms
S.tilde.naive <- 1/s2.tilde.naive * (t(X) %*% y - t(X) %*% X %*% beta.tilde)
S.bar.naive <- 1/s2.bar.naive * (t(X) %*% y - t(X) %*% X %*% beta.bar)
U.naive <- R2 %*% solve(W) %*% (S.tilde.naive - S.bar.naive)
Score.U.naive <- 1/(N*s2.hat.naive) * t(U.naive) %*%
  solve(R2 %*% solve(W) %*% t(R2)) %*% U.naive
1 - pchisqbar(Score.U.naive, df = 0:h, wt.bar = wt.bar)
1 - pfbar(Score.U.naive, df1 = 0:h, df2 = nrow(X)-ncol(X), wt.bar = wt.bar)

# Score.null-info.corr using corrected unit information matrix and corrected mean
# squared error terms
# Silvapulle & Silvapulle (1995), p. 342
Score.nullinfo.corr <- 1/N * c(S.bar.corr - S.tilde.corr) %*% solve(I.bar.corr) %*%
  (S.bar.corr - S.tilde.corr)
1 - pchisqbar(Score.nullinfo.corr, df = 0:h, wt.bar = wt.bar)
1 - pfbar(Score.nullinfo.corr, df1 = 0:h, df2 = nrow(X)-ncol(X), wt.bar = wt.bar)
```

```r
# Score.null-info.naive using naive unit information matrix and naive mean squared
# error terms
Score.nullinfo.naive <- 1/N * c(S.bar.naive - S.tilde.naive) %*%
  solve(I.bar.naive) %*% (S.bar.naive - S.tilde.naive)
1 - pchisqbar(Score.nullinfo.naive, df = 0:h, wt.bar = wt.bar)
1 - pfbar(Score.nullinfo.naive, df1 = 0:h, df2 = nrow(X)-ncol(X), wt.bar = wt.bar)

# Score.inf.corr using corrected unit information matrix
# Silvapulle & Sen (2005), p. 166
P.corr <- R2 %*% solve(I.hat.corr) %*% (S.tilde.corr-S.bar.corr)
Score.info.corr <- 1/N * t(P.corr) %*% solve(R2 %*% solve(I.hat.corr) %*%
                                          t(R2)) %*% P.corr
1 - pchisqbar(Score.info.corr, df = 0:h, wt.bar = wt.bar)
1 - pfbar(Score.info.corr, df1 = 0:h, df2 = nrow(X)-ncol(X), wt.bar = wt.bar)

# Score.inf.naive using naive unit information matrix
P.naive <- R2 %*% solve(I.hat.naive) %*% (S.tilde.naive-S.bar.naive)
Score.info.naive <- 1/N * t(P.naive) %*% solve(R2 %*% solve(I.hat.naive) %*%
                                            t(R2)) %*% P.naive
1 - pchisqbar(Score.info.naive, df = 0:h, wt.bar = wt.bar)
1 - pfbar(Score.info.naive, df1 = 0:h, df2 = nrow(X)-ncol(X), wt.bar = wt.bar)

# Score.VCOV
V <- R2 %*% VCOV %*% (S.tilde.corr-S.bar.corr)
Score.VCOV <- t(V) %*% solve(R2 %*% VCOV %*% t(R2)) %*% V
1 - pchisqbar(Score.VCOV, df = 0:h, wt.bar = wt.bar)
1 - pfbar(Score.VCOV, df1 = 0:h, df2 = nrow(X)-ncol(X), wt.bar = wt.bar)
```
