# Supplementary material for: Overview and evaluation of various frequentist test statistics using constrained statistical inference in the context of linear regression
Source: Front Psychol. 2022 Oct 14;13:899165. doi: 10.3389/fpsyg.2022.899165 (PMC9614349; doi:10.3389/fpsyg.2022.899165)
Supplement: Supplementary file 5 [file Data_Sheet_5.PDF]

Translating a linear regression problem into a quadratic program

For OLS estimation, we minimize the sum of the squared residuals to find a solution:

$$\begin{aligned} SSE_{OLS} &= \sum_{i=1}^{n} \hat{e}_i^2 \\ &= \sum_{i=1}^{n} (y_i - \hat{y}_i)^2. \end{aligned}$$

In matrix notation, SSE can be re-written to:

$$\begin{aligned} SSE_{OLS} &= (\boldsymbol{y} - \hat{\boldsymbol{y}})'(\boldsymbol{y} - \hat{\boldsymbol{y}}) \\ &= \boldsymbol{y}'\boldsymbol{y} - \boldsymbol{y}'\hat{\boldsymbol{y}} - \hat{\boldsymbol{y}}'\boldsymbol{y} + \hat{\boldsymbol{y}}'\hat{\boldsymbol{y}} \\ &= \boldsymbol{y}'\boldsymbol{y} - 2 \times \boldsymbol{y}'\hat{\boldsymbol{y}} + \hat{\boldsymbol{y}}'\hat{\boldsymbol{y}} \\ &= \boldsymbol{y}'\boldsymbol{y} - 2 \times \boldsymbol{y}'\boldsymbol{X}\boldsymbol{\beta} + (\boldsymbol{X}\boldsymbol{\beta})'\boldsymbol{X}\boldsymbol{\beta} \\ &= \boldsymbol{y}'\boldsymbol{y} - 2 \times \boldsymbol{y}'\boldsymbol{X}\boldsymbol{\beta} + \boldsymbol{\beta}'\boldsymbol{X}'\boldsymbol{X}\boldsymbol{\beta}. \end{aligned}$$

Since the first term $\boldsymbol{y}'\boldsymbol{y}$ does not depend on $\boldsymbol{\beta}$, it can be removed:

$$SSE_{OLS} = -2 \times \boldsymbol{y}'\boldsymbol{X}\boldsymbol{\beta} + \boldsymbol{\beta}'\boldsymbol{X}'\boldsymbol{X}\boldsymbol{\beta}.$$

`solve.QP()` uses $\frac{1}{2}$ for its quadratic term, so we have to divide by 2:

$$SSE_{qp} = -\boldsymbol{y}'\boldsymbol{X}\boldsymbol{\beta} + \frac{1}{2} \times \boldsymbol{\beta}'\boldsymbol{X}'\boldsymbol{X}\boldsymbol{\beta}.$$

This results in the kind of quadratic programming problem used by `solve.QP()`.
